# Agent-Based Learning Model for the Obesity Paradox in RCC

**DOI:** 10.3389/fbioe.2021.642760

**Published:** 2021-04-29

**Authors:** Matteo Belenchia, Giacomo Rocchetti, Stefano Maestri, Alessia Cimadamore, Rodolfo Montironi, Matteo Santoni, Emanuela Merelli

**Affiliations:** ^1^Laboratory of Data Science and Bioshape, School of Science and Technology, University of Camerino, Camerino, Italy; ^2^Centre de Physique Théorique, Aix-Marseille University, Marseilles, France; ^3^Section of Pathological Anatomy, Polytechnic University of the Marche Region, School of Medicine, United Hospitals, Ancona, Italy; ^4^Department of Oncology, Macerata Hospital, Macerata, Italy

**Keywords:** renal cell carcinoma, immunotherapy, multiagent system, interaction-as-perception paradigm, bioagent, computational biology, dynamical networks, cell-cell interaction network

## Abstract

A recent study on the immunotherapy treatment of renal cell carcinoma reveals better outcomes in obese patients compared to lean subjects. This enigmatic contradiction has been explained, in the context of the debated obesity paradox, as the *effect* produced by the cell-cell interaction network on the tumor microenvironment during the immune response. To better understand this hypothesis, we provide a computational framework for the *in silico* study of the tumor behavior. The starting model of the tumor, based on the cell-cell interaction network, has been described as a multiagent system, whose simulation generates the hypothesized effects on the tumor microenvironment. The medical needs in the immunotherapy design meet the capabilities of a multiagent simulator to reproduce the dynamics of the cell-cell interaction network, meaning a reaction to environmental changes introduced through the experimental data.

## 1. Introduction

The obesity paradox refers to the fact that although obesity is a risk factor for developing clear cell renal cell carcinoma (ccRCC or simply RCC), it is found to be associated with a more favorable prognosis. In other words, obese patients who undergo treatment with RCC immunotherapy survive longer than those with normal weight and the same advanced cancer.

This long-known paradox has recently gained attention thanks to the work of Sanchez et al. (2019) describing *the effects* of the immunotherapy treatments on the RCC microenvironment. The authors studied the *immune response* both in lean and obese patients with RCC, by investigating the *angiogenic and immunological transcriptomic profiles* of the tumor and the *perinephric adipose tissue*, that is the adipose tissue immersed in the renal microenvironment.

**Figure 1:**
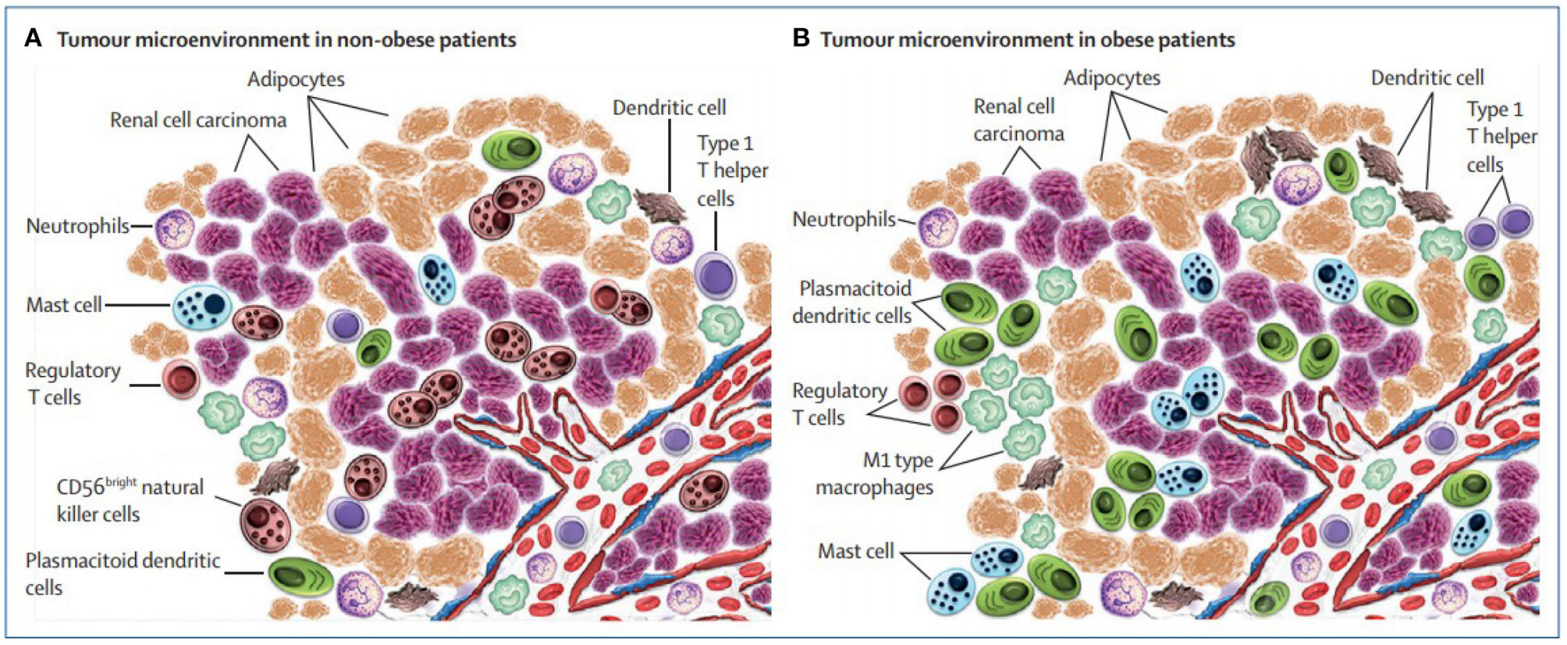
Microenvironment surrounding the tumor in non-obese vs. obese patients with renal cell carcinoma (picture courtesy of Santoni et al., 2019).

The most relevant finding was that the inflammation of peritumoral adipose tissue increased in obese patients with body mass index (BMI)>30 kg/m^2^^1^ especially close to the tumor. The interplay between the *tumor* and *peritumoral adipose tissue* might have clinical relevance and give account for the paradox (see Figure 1) as discussed and depicted in Santoni et al. (2019).

To support this conjecture, it is natural to think of modeling the RCC system with a formal approach that allows to describe the knowledge of the oncologists and acquiring new knowledge coming from experimental data. Given the characteristics of the RCC system, the model must be able to represent the microenvironment as the main component in which immune and tumor cells are spatially distributed and move influenced by blood vessels, and whose effect on the tumor is function of BMI.

The proposed approach aims to overcome the limitation of several related studies proposed in the literature, most of them exploiting mathematical models based on ordinary differential equations and partial differential equations enriched with stochastic elements, as well as other physical models based on complex networks and phase transitions analysis (Davies et al., 2011); however, none of them allow the explicit description of the environment as a main component of the model.

**Figure 2:**
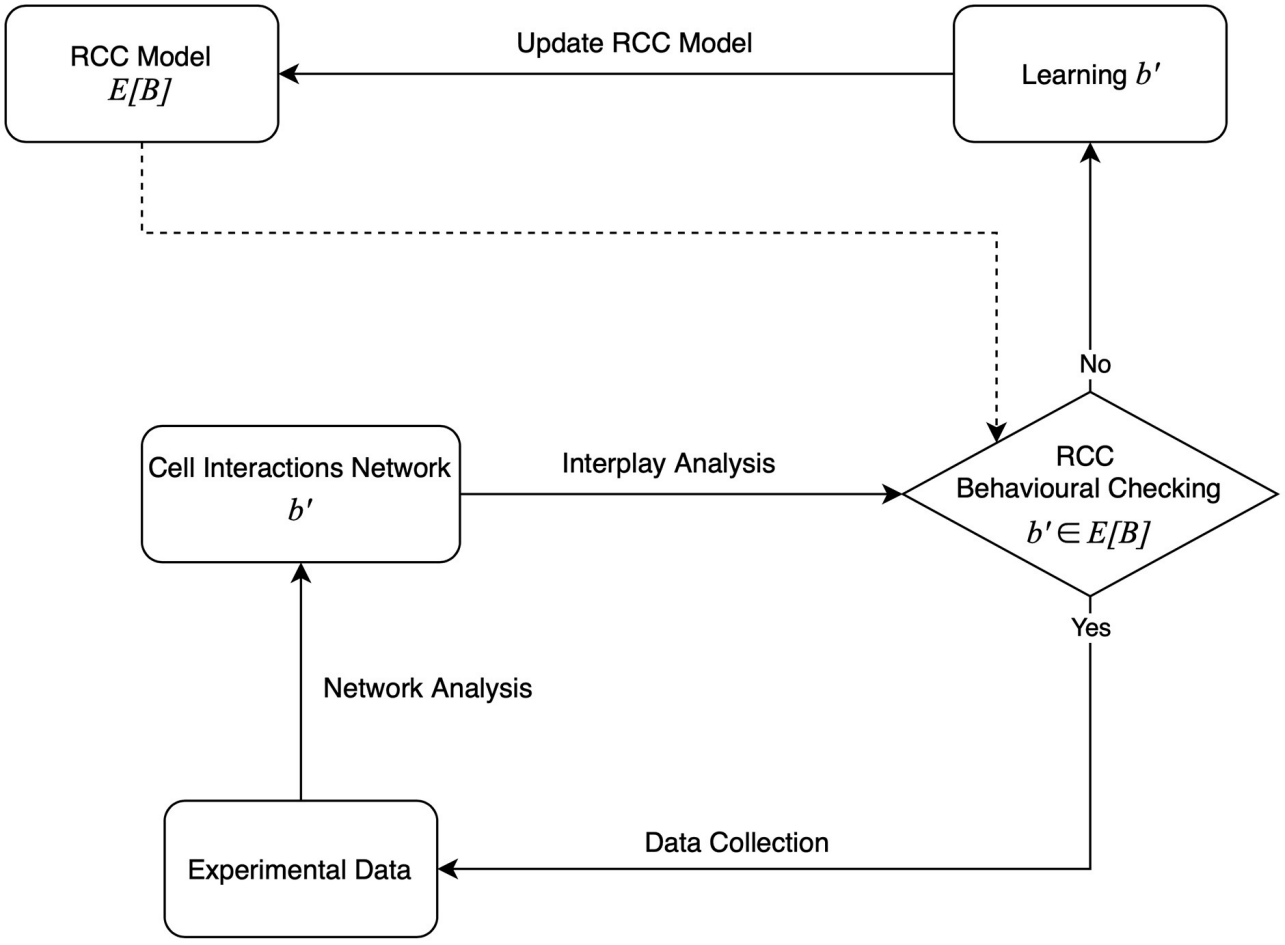
The agent-based learning model.

In this work, we introduce a theoretical framework suitable to support the learning process through which the RCC model is dynamically defined during the immunotherapy treatment delivered to different patients (Figure 2). The method is inspired by our previous works (Bartocci et al., 2007; Merelli et al., 2015).

The model is based on the agent paradigm, consisting of a collection of interacting autonomous components and the *environment* where they *live* (Merelli et al., 2007). It describes a self-organized system able to react to changes in the environmental parameters. The resulting behavior, the immune response, can be explained in terms of emerging properties. The RCC model updates when the immune response changes the interaction matrix; this can be viewed as a learning process.

*The immune response* is a property of the peritumoral tissue whose structure results from the group of cell agents, immune and tumor cells, interacting in the tumor microenvironment. We aim to define a model of a system whose behavior is function of the fat concentration in the environment; for this reason, we do not take into account the behavior of the adipocytes, but only their quantity.

Several studies already exploited the agent-based approach to model the tumor microenvironment, in some cases as part of a multiscale setting for providing *in silico* support to drug and therapy design (Yankeelov et al., 2016; Norton et al., 2019). An *agent* represents an active component of the system, such as an immune or a tumor cell, whereas *the environment* is the representation of the peritumoral and intratumoral environment. In our work, *the emerging properties* are observable from the RCC model simulation, whose dynamics is expressed following the *interaction-as-perception* paradigm (Piangerelli et al., 2020) declined in this context: whenever a cell agent perceives, another compatible cell agent *moves toward* to interact with it, activating or inhibiting the production of compatible cell agents. All the agents' interactions that characterize the immune response are a dynamic representation of the cell-cell interaction network bounded by the tumor environment and regulated by the obesity index BMI modeled as a parametrized 3D space.

## 2. Biological and Theoretical Background

A tumor is an abnormal agglomerate of cells having lost their ability to regulate the expression of their genetic information correctly, and thus generally increase their number in an uncontrolled manner, as opposed to normal cells. As the tumor cells duplicate and its mass get larger, it will eventually require more oxygen and nutrients than it can receive; to meet this need, the tumor creates new blood vessels in a process called *angiogenesis*. After that, the tumor can grow faster and it can spread through the blood to other tissues and organs in the body.

A tumor grows in a surrounding environment (tumor microenvironment) made by other cells, among which we can identify different types of immune cells. Moreover, in the kidneys and other organs, we can observe the presence of adipocytes (or fat cells). The adipocytes near the tumor microenvironment form the peritumoral fat.

The formation of tumor cells triggers the immune response, which aims to eliminate the mutated cells before they reach, by multiplying themselves exponentially, a number too large to be contained.

The models described in this article are based on the experimental evidence provided by Sanchez et al. (2019) and Santoni et al. (2019) on a kidney cancer, the RCC; in accordance with these studies, we selected a subset of the immune cell types identified in the RCC microenvironment and peritumoral fat, and specified an agent-based representation of their properties and interactions.

In our study, we consider as immune cell types the *T cells* (CD4+ and CD8+ naive, CD8+ cytotoxic, Regulatory, CD4+ helper 1 and 2), the *dendritic cells* (DCs) [conventional DCs (cDCs) and plasmacytoid DCs (pDCs)], the *macrophages* (M1 and M2 phenotypes), the *mast cells*, the *natural killers* (NKs), and the *neutrophils*.

An overview of the key functions they carry out in living systems is provided in Table 1; for a more comprehensive description of each of them, correlated with the cell models generated basing on their properties, refer to the Supplementary Information.

**Table 1:** Overview of the cell types taken into account in our models and the key functions they carry out in the reaction against the tumor (in living systems).

**Cell type**	**Subpopulation**	**Description**
T cells	CD4+ naive	Immature cells that can respond to novel pathogens, which the immune system has not yet encountered; however, they need to be activated by an antigen-presenting cell (APC). If activated, they differentiate into CD4+ helper 1 T cell or CD4+ helper 2 T cell.
	CD4+ helper 1	T cell lineage that carries out an anti-tumor activity, involving the activation of dendritic cells (Mailliard et al., 2002) and of the M1 macrophages, and the direct kill of the tumor cells through the TNF-related apoptosis-inducing ligand (TRAIL) pathway (Lin and Karin, 2007).
	CD4+ helper 2	Subtype of helper T cells that attract tumor-specific CD8+ cytotoxic T cells and activate the M1 macrophages (Carretero et al., 2015). Additionally, the presence of helper 2 T cells is associated with tumor proliferation (Kim and Cantor, 2014).
	CD8+ naive	They represent the naive form of CD8+ cytotoxic T cells. Like their CD4+ counterpart, they can react to novel pathogens, but need to be activated by an APC.
	CD8+ cytotoxic	White blood cells able to destroy cancer cells and other types of damaged cells.
	Regulatory (Treg)	They suppress or downregulate T cells' proliferation. In RCC, Treg cells are involved in tumor development and progression by inhibiting anti-tumor immunity.
Dendritic cells	Conventional (cDCs)	Antigen-presenting cells whose main function is to present antigens to both CD4+ helper T cells and CD8+ cytotoxic T cells.
	Plasmacytoid (pDCs)	Major producers of type I interferon (IFN type I), an important immune system activity regulator (Koucký et al., 2019).
Macrophages	M1 Phenotype	White blood cells that ingest (phagocyte) substances and cells external or harmful to the organism, including microbes and cancer cells. M1 macrophages also enhances CD8+ cytotoxic T cells anti-tumor activity (Vlahopoulos, 2017) and promotes CD4+ helper 1 T cell differentiation (Lin and Karin, 2007).
	M2 Phenotype	Macrophage phenotype able to moderate the inflammatory response and stimulate angiogenesis and tumor growth (Dandekar et al., 2011).
Mast cells	–	Immune cells that are able to activate the dendritic cells. In the case of the RCC, it has been observed by Chen et al. (2017) that they foster tumor angiogenesis.
Natural killers	–	Cytotoxic lymphocytes that are able to kill tumor cells, even in the absence of surface antigens.
Neutrophils	–	First type of inflammatory cells that move toward the site of an inflammation. According to Santoni et al. (2019), the prevalence of neutrophils was observed to be unchanged in both lean and obese patients.

We model the tumor microenvironment as a collection of agents (or BioAgents), which, by acting and interacting with one another, give origin to a complex network. However, the concept of agent cannot be separated from that of the *environment*, which is part of the agent-based approach. The environment is a first class component, which means that it has its own identity.

The model defined this way is the basis of an agent-driven simulation, in which the biological information is approximated through set of variables and parameters. For this reason, experimental data, such as the initial cell concentrations or the time at which a treatment starts, are needed to setup the simulation environment. In this context, the peritumoral fat is an environmental parameter, which affects the agent interactions.

The simulation is implemented over a software platform; in our case, we rely on Repast Simphony, a Java-based modeling system for interacting agents. This simulation tool establishes some constraints we had to meet in the definition of the software counterpart of each biological component.

**Figure 3:**
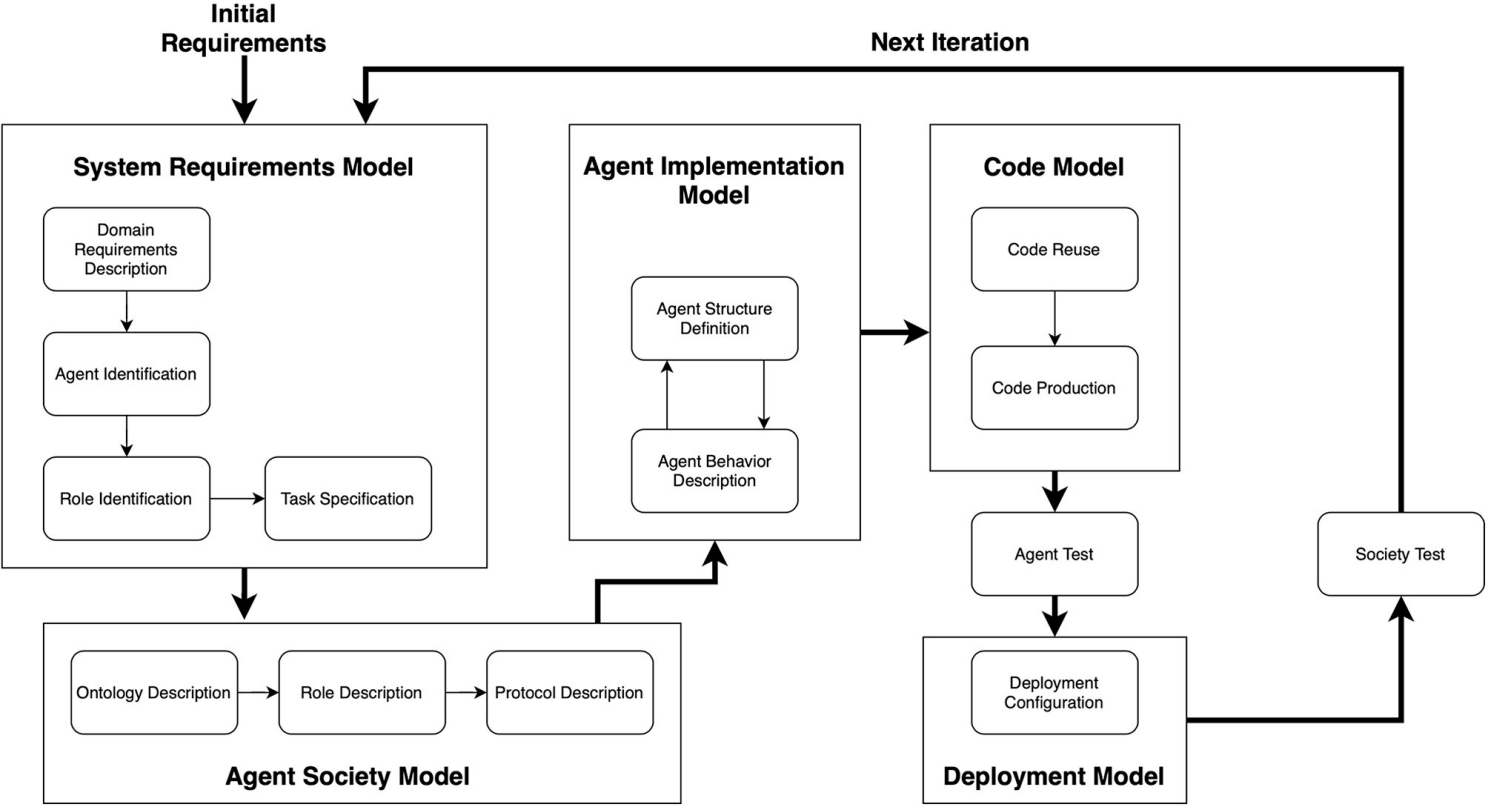
An overview of the PASSI methodology. It is composed of five phases, each one producing a specific model. Image adapted from Cossentino (2005).

For the purpose of this paper, the agent-based modeling did not require a mathematical description of its components, because, for engineering the model on the Repast framework, it is sufficient to describe the agent behavior by using a meta-model. For this reason, the system has been designed following the PASSI (Process for Agent Societies Specification and Implementation) methodology (Cossentino, 2005) (see Figure 3). In the Supplementary Information, we provide the diagrams that describe the agents' behavior and some of their interactions.

Most notably, we needed to represent the tumor microenvironment as a 3D cube, whose volume should be set before launching the simulation. However, this representation of the simulation environment should not be confused with the concept of collection-of-agents described above. A simulated structure is an approximation of a biological component that uses the agent-oriented approach to express its information.

In the rest of this section, we will go further into the details of the modeling and simulation choices we made to carry out our studies.

## 3. Materials and Methods

### 3.1. Agent-Based Model of RCC

We study the obesity paradox as a behavior emerging from the local interactions characterizing the RCC microenvironment. For this reason, we defined an agent-based model of the dynamics involved in the immune response to RCC, as a reaction triggered by the beginning of an immunotherapy treatment.

As deepened in the rest of this section, the cell types listed above are abstracted as the categories of bioagents of our system. The model constructed this way has been implemented as a multiagent simulator (OncoAgent) based on the Repast Simphony execution environment.

#### Emerging Properties of the Immune System

In the RCC microenvironment, there are a number of pathways in action, new proteins being produced, and the proteins themselves are also involved in signaling to other proteins or cells, in a cascade of causes and effects. In this regard, it has been considered more appropriate and suitable to directly model the effects of these pathways on the environment, by selecting those considered to be of importance in our study. In our preliminary studies, all these interactions have been reduced to a list of *13 effects* that can, in any combination, influence the behavior of an agent.

Any agent can be the source of one or more effects, and each agent collects effects from other agents inside a fixed radius. To maintain the locality of the effects, the value of this radius must be kept fairly small, and by default has been set to 30μ*m*.

Each effect is represented by an integer, which can be positive or negative, whether it increases or decreases the occurrence rate of an event; when it has no impact on the event, it is equal to 0. If an agent does not produce any of the 13 possible effects, equivalently means that it produces an effect with value 0.

At each step of the simulation based on this model, after the time step at which the RCC immunotherapy starts, every agent collects, inside the specified radius, the effects from which its behavior can be influenced.

Once all the effects are collected, they are summed up componentwise and the resulting values, a sort of probabilities, when relevant to the agent, are used to influence its actions. Thus, these actions are subjected to a probability, as better described in section 2 of the Supplementary Information.

Additionally, in the model representing obese individuals, the environment can expose effects. These effects are as follows: M1 Macrophage Mutation Effect, M2 Macrophage Mutation Effect, Natural Killer Cell Kill Rate Effect, and Regulatory T Differentiation Effect.

All the 13 effects are summarized in Table 2. New effects can be added easily into the system, so this list is by no means final and may be expanded in future works.

**Table 2:** The effects modeled in the system.

**Effect**	**Description**
Angiogenesis Effect	Affects the likelihood for the tumor as a whole to start angiogenesis.
Cytotoxic T Cell Activation Effect	Affects the likelihood to successfully activate a CD8+ naive T cell into a CD8+ cytotoxic T cell.
Cytotoxic T Cell Apoptosis Effect	When positive, enables a chance of a CD8+ cytotoxic T cell to undergo apoptosis (cell death); the greater the value, the greater the likelihood.
Cytotoxic T Cell Kill Rate Effect	Affects the likelihood for a CD8+ cytotoxic T cell to eliminate a tumor cell.
Cytotoxic T Cell Proliferation Effect	Affects the rate of duplication of CD8+ cytotoxic T cells.
Dendritic Cell Phagocytosis Effect	Affects the likelihood for a dendritic cell or plasmacytoid dendritic cell to phagocyte a tumor cell.
M1 Macrophage Mutation Effect	Affects the rate of the M1 macrophages switching into the M2 phenotype.
M2 Macrophage Mutation Effect	Affects the rate of the M2 macrophages switching into the M1 phenotype.
Natural Killer Cell Kill Rate Effect	Affects the likelihood for a natural killer cell to eliminate a tumor cell.
Regulatory T Differentiation Effect	Affects the rate of CD4+ naive T cells differentiation into regulatory T cells.
Helper 1 T Proliferation Effect	Affects the rate of duplication of CD4+ helper 1 T cells.
Tumor Apoptosis Effect	When positive, enables a chance of a tumor cell to undergo apoptosis (cell death); the greater the value, the greater the likelihood.
Tumor Growth Effect	Affects the rate of duplication of RCC tumor cells.

#### The Categories of BioAgents

In our agent-based model, each cell, whether it belongs to the tumor or the immune system, is represented by an agent.

As introduced previously in this section, we identified six immune cell types as the main BioAgent categories of our model. Some of these cell types are represented in the form of specific subpopulation, which are the biological counterpart of the category instances.

They can perform defined actions and present specific *effects* to other agents. In Table 3, we introduce the functions modeled for each cell type (a complete explanation is provided in the Supplementary Information).

**Table 3:** Overview of the BioAgent categories, and the related behaviors, that we derived from the immune cell types taken into account in our models.

**BioAgent**	**Behavior**	**Description**
T cells	CD4+ naive	They have an associated bit string of length 1 byte that represents their antigen receptors. It is activated with the same mechanism at the core of the Celada–Seiden model for antigen recognition (Celada and Seiden, 1992). In case of activation, the agent differentiate into either CD4+ helper 1 T cell or CD4+ helper 2 T cell, with equal probability.
	CD4+ helper 1	They perceive and move toward the tumor mass and have a chance to activate new dendritic cell and M1 macrophage agents that appear at the edge of the environment. They additionally have a chance to proliferate.
	CD4+ helper 2	They activate new M1 macrophage agents and attract CD8+ cytotoxic T cell agents. Can also proliferate.
	CD8+ naive	Like their CD4+ counterpart, they have a bit string, 1 byte long. In case of successful activation, the agent turn into a CD8+ cytotoxic T cell, otherwise it is removed and then recreated anew in a different location with a new bit string.
	CD8+ cytotoxic	They perceive and move toward RCC tumor cell agents and attempt to destroy them. They can proliferate.
	Regulatory (Treg)	They perceive and move toward the tumor mass where they exposes a different suppressor effects.
Dendritic cells	Conventional (cDC)	They perceive and move toward RCC tumor cell agents and attempt to phagocyte them. They also attempt to activate the closest naive T cell.
	Plasmacytoid (pDC)	This agents have a chance to activate new natural killer cell agents, which are added into the environment at one of its edges. They may generate a combination of the different effects, the most important of which is the angiogenesis effect.
Macrophages	M1 Phenotype	They perceive and move toward RCC tumor cell agents and phagocyte them. Also try to activate CD4+ naive T cell agents. They might undergo a phenotype switch and become a M2 macrophage, influenced by the M1 macrophage mutation effect.
	M2 Phenotype	Like their M1 counterpart, they might switch to the other phenotype influenced by the M2 macrophage mutation effect. They generate positive angiogenesis effect and tumor growth effect.
Mast cells	Mast cell	They perceive and move toward the tumor mass and have the chance of activating new dendritic cell agents that appear at the edge of the environment. They may produce a combination of the various effects, including the angiogenesis effect.
Natural killers	Natural killer	They perceive and move toward RCC tumor cell agents and attempt to destroy them, influenced by the Natural Killer Cell Kill Rate Effect.
Neutrophils	Neutrophil	Dummy agents, not performing any action nor producing any effect.

*A brief description of their behavior is also provided (for a comprehensive explanation, refer to the Supplementary Information)*.

The *RCC tumor cell agents* represent the category against which the action of the immune cell agents is directed. They have a certain chance to duplicate, defined by a parameter. This possibility is influenced by the Tumor Growth Effect and by whether or not the tumor mass, as a whole, has undergone angiogenesis. The chance of duplicating is doubled in case of non-negative Tumor Growth Effect, while mildly improved by a negative effect. Additionally, the agent might also experience apoptosis: this happens when it receives a Tumor Apoptosis Effect greater than zero.

Angiogenesis can happen if there is a RCC tumor cell agent close enough to the environment's blood vessel; it is determined by the Tumor Angiogenesis Effect. In case of success, a new blood vessel, connecting the selected agent to the environment's blood vessel, is added by means of an uninterrupted sequence of blood agents. Angiogenesis can happen multiple times, and different new blood vessels can be added, although the effect on tumor growth is not affected by that. However, the presence of multiple blood vessels makes it less likely that the immune system can disrupt the flow of oxygen and nutrients.

At each step of the simulation based on our models, it is determined which RCC tumor Cell agents have access to the blood supply. This is done by taking a list of all the agents that triggered angiogenesis (which are considered to have access to the blood) and then verifying, for any other RCC tumor cell, if there exists a path that connects them to any member of the list. This means that the access to the bloodstream is granted for any contiguous set of RCC tumor cell agents that contains one of the agents triggering angiogenesis. The algorithm used for this process is a Breadth-First Search.

#### The Tumor Microenvironment

The tumor microenvironment has been modeled by taking into account two key features: the presence of *blood vessels* and the *BMI* of the patient.

The blood vessels have many important roles in the RCC micro-environment, as they are first and foremost the channel from which white blood cells can reach the neoplastic cells they seek to eliminate. This particular feature has not been modeled directly, but it is implicitly represented by the fact that new immune system cells appear from outside the boundaries of the modeled environment, behaving just as an open system. The other important role that instead was directly modeled is their function as suppliers of the much needed oxygen, which enhances the tumor cells growth. A blood vessel is built up from *blood* agents, which have no other function besides being a visual representation of where the blood vessel is located. This particular blood vessel can be the target of tumor angiogenesis, having important effects on tumor growth and spread (Nishida et al., 2006): the tumor can create new blood vessels that attach to this target and increase its rate of growth and spread to other tissues through blood, although this last part was not implemented because outside of the scope of the simulation.

The BMI is a very simple measure of body fat based on height and weight. The effects of the BMI in the case of RCC become evident when its value is ≥ 30 *kg*/*m*^2^ (Santoni et al., 2019), and in that case it was decided to model the environmental effect of obesity by reproducing the leptin hormone effects on the immune system. Leptin signaling has various functions (Naylor and Petri, 2016), and of these two were modeled: the suppression of regulatory T cells (Treg) differentiation and the impairment of NK cells cytotoxicity. Moreover, it was observed by Castoldi et al. (2016) that adipose tissue in obese individuals present higher numbers of M1 macrophages, while in lean humans and mice it is the M2 macrophages that predominate. This last effect was modeled by increasing the rate of M2 macrophages switching to the M1 phenotype while also decreasing the rate of switching in the opposite direction in simulations regarding obese individuals. Finally, the BMI also determines the number and proportion of each type of immune system cells at the start of the simulation, following the guidelines in Santoni et al. (2019). In particular, the total number of agents in case of obese and non-obese simulations is the same (initially), as well as the number of T cells, while the proportion of the other agents is changed as it was observed in the study. The change of proportion concerns *NK cell, mast cell*, and pDC agents. Lean individuals have a higher proportion of NK cells and lower proportion of mast cells and pDCs.

#### The Peritumoral Adipose Tissue

Sanchez and colleagues reported that obese individuals have a higher number of DCs, M1 macrophages, and T cells in the adipose tissue around the tumor (Sanchez et al., 2019), as shown in **Figure 5**; we modeled these findings by adding an extra quantity of the related immune cell agents for simulating the tumor microenvironment of obese individuals. This value is defined for each cellular species at the beginning of the simulation and can be set according to the experimental data.

For our preliminary studies, we placed an arbitrary, although reasonable, additional amount of the aforementioned cell types, picked at random, in the simulated obese microenvironment. This choice is justified by the fact that not all agents in the system play a measurable role in the fight against cancer, as they are randomly placed and might be too far away, but some of these additional agents, placed close enough to the fat near the tumor, might have a relevant impact. In this way, we model the possibility that some of the immune cells located in the fat might perceive and move onto the tumor mass and contribute to a positive outcome.

### 3.2. The Simulation Environment

The simulation environment is a cube, whose size is set by the user. Its edges are not connected and it is not possible for the agents to exit its volume. It is organized in a grid, whose blocks are multi-occupancy, meaning that multiple agents can coexist in a single block. In the environment, the various agents that represent the cells of the innate and adaptive immune systems are placed randomly at the start of the simulation. Differently, a single RCC cell agent is placed at a distance from the blood vessel that is decided by the user before the beginning of the simulation. All agents, except for the RCC cell agents, do not act at all until a later step, which represents the starting of the immunotherapy treatment that enables an immune response. This step is also established by the user and inserted as a parameter.

**Figure 4:**
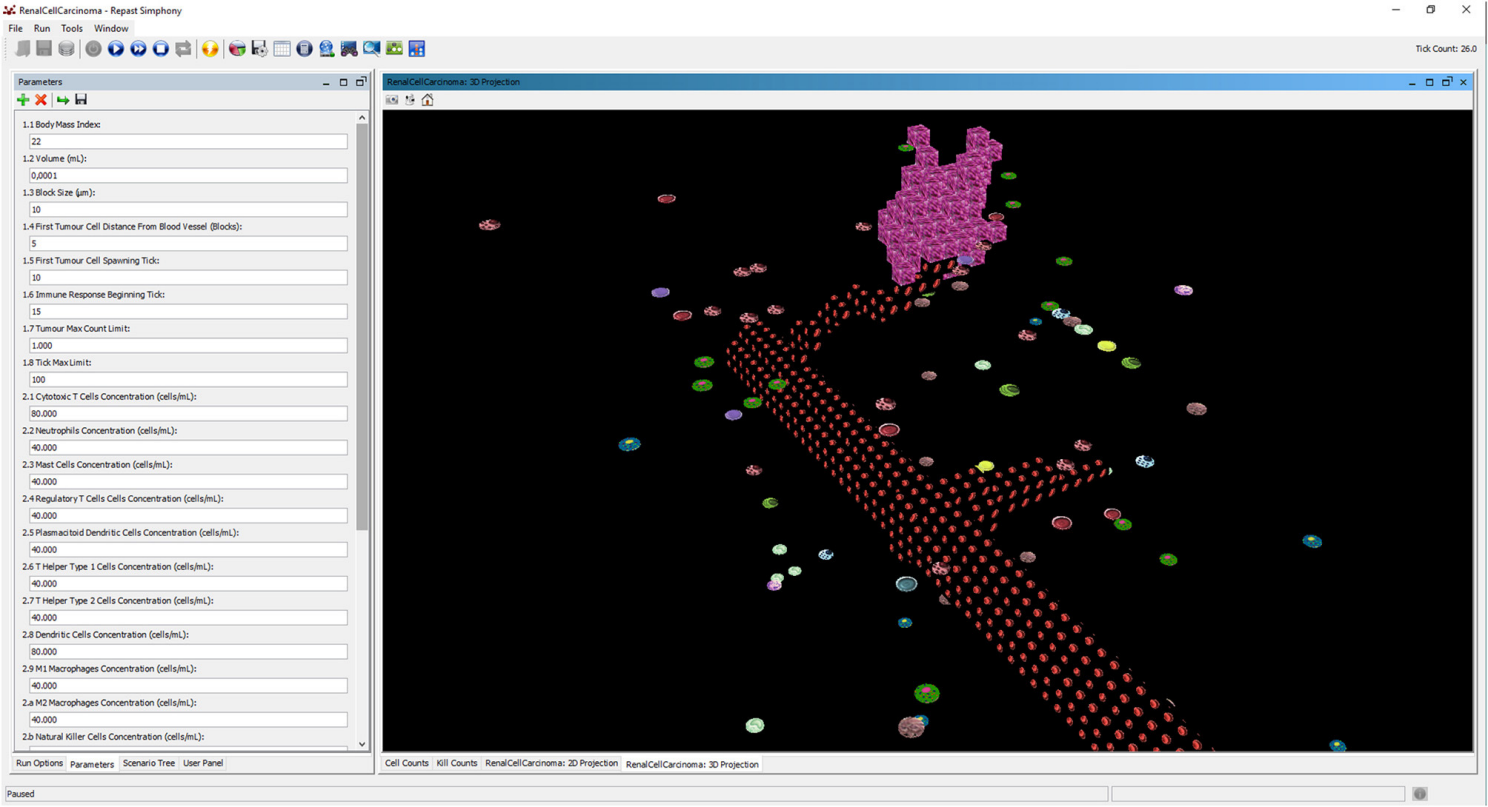
The interface of repast simulator with the list of configuration parameters and the 3D display of the runtime dynamics during the immunotherapy treatment.

All the parameters required to run the simulation are set through the *Parameters tab* of the repast runtime interface, as shown in Figure 4. All the parameters accessible from this interface are listed in **Tables 5**, **6**.

#### Termination of the Simulation

The conclusion of a simulation entails three possible outcomes, each determined by a specific termination condition. Any of these conditions, if verified, triggers the end of the simulation.

1. If the simulation elapses past a certain time step (set by the user), we have an *inconclusive outcome*: neither the tumor nor the immune system prevailed on the other. It can be interpreted as a condition where the tumor growth is somewhat contained by slowing down its exponential expansion to prevent an excessive proliferation.
2. If the number of tumor cells proliferate past a certain, large, amount (also established by the user), the simulation ends with a *tumor proliferation outcome*. In this case, the tumor is considered unstoppable and prognosis for the patient is poor.
3. If the simulation reaches a point in which all the RCC cell agents have been eliminated, we obtain a *tumor remission outcome*. In this case, the tumor has been healed; however, this result refers to a specific tumor mass and does not mean that the patient has completely recovered from cancer.

#### Simulation Interface and Output

**Figure 5:**
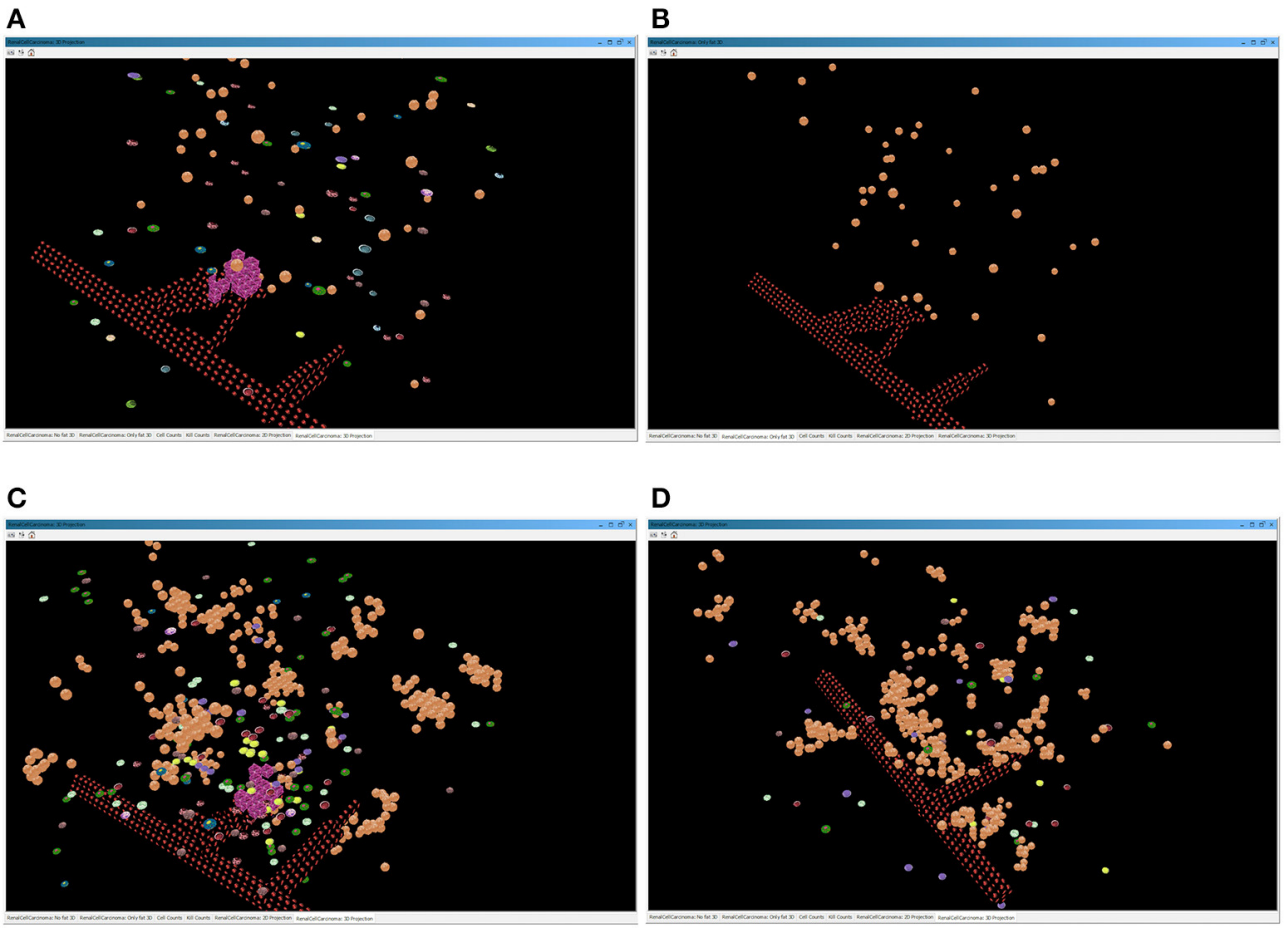
Screenshots of the simulator interface, showing the progression of the simulation in the virtual 3D environment. The two images above represent a simulation of a lean patient with BMI = 22. **(A)** All the cells present in the tumor environment during the immunotherapy treatment; **(B)** only the graphical representation of the adipocytes immerse in the renal microenvironment, with no evidence of immune cells. The two images below depict a simulation of an obese patient with BMI = 35. In **(C)**, the layer of BioAgents shows all the cells present in the tumor environment during the immunotherapy treatment; in **(D)**, the layer represents only the adipocytes cells and the immune cells due to the inflammation that contribute to the immune response.

The progression of a simulation can be visualized using a 3D projection as shown in the interface screenshot in Figure 4. Moreover, the simulator can visualize different layers of the BioAgents populating the tumor environment, such as the layer of the microenvironment as a whole, or the layer representing only the adipose tissue immerse in the renal microenvironment (see Figure 5). Because the current model considers adipocytes only from a quantitative perspective, they are represented in the 3D environment just graphically, meaning that they do not have any active role. We adopted a similar approach to provide a visual representation of the bloodstream and show the formation of new blood vessels during angiogenesis.

**Figure 6:**
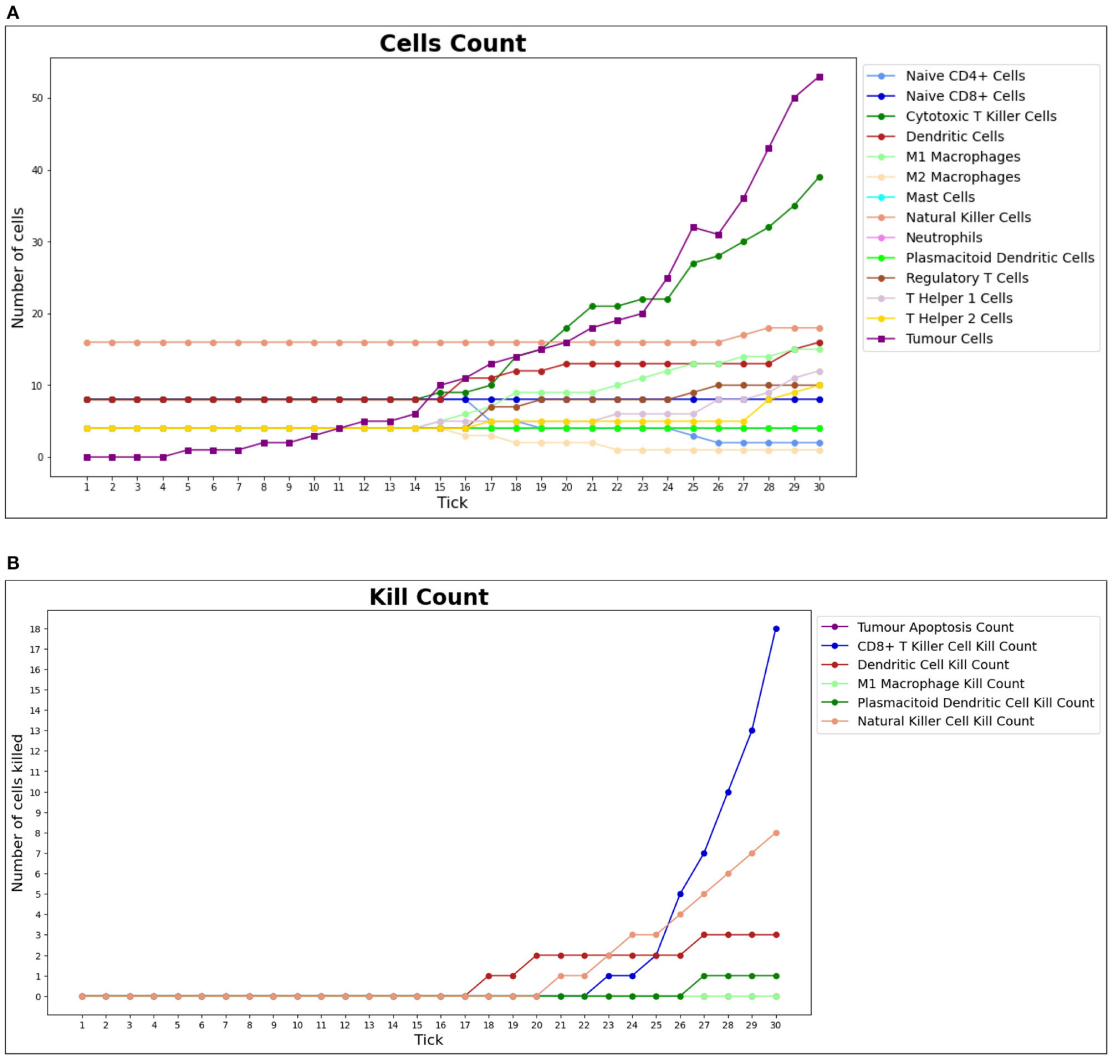
Cell counts and Kill counts for a sample run. **(A)** The number of bioagents by cell type at each step (tick) of a sample simulation for a lean individual. At tick 10, the first tumor cell appears and starts duplicating, while the immune system response begins at tick 15: most notably NK cells and T killer cells soar in reaction to the therapy and tumor growth is hampered. In **(B)**, the number of tumor cells eliminated by each type of bioagent, calculated cumulatively at each step (tick) of a sample simulation for a lean individual. The tumor apoptosis data shown is the number of tumor cells that successfully underwent apoptosis as a result of the effects of T helper 1 cells. It can be noted that most of the elimination work is done by NK cells and T killer cells.

In the course of a simulation run, it is also possible to inspect two time series charts: the *cell counts* (Figure 6A) and the *kill counts* (Figure 6B). The cell counts chart shows the number of agents by type for each step of the simulation. The kill counts chart represents the number of RCC cell agents eliminated by immune cell agents (displayed basing on agent types) and by the tumor apoptosis effects.

For each simulation, three comma separated values (CSV) files are produced. In all of them, every line reports determined values, corresponding to the state of the simulated environment at a specific tick of the simulation clock. The first two are the CSV representations of the charts described in the previous paragraph. The last one is the *outcome file*. It shows the results of an individual simulation that can be one of the three outcomes described in the previous section.

Each CSV file also reports the random seed of the simulation, so it can be repeated, and the run number, which is useful in the case of batch runs, as explained in the next section.

### 3.3. Study Phases

We carried out our study in two phases:

1. a verification phase, in which we tested the effectiveness of our simulation approach basing on rough data derived from the information provided by Sanchez et al. (2019) and Santoni et al. (2019);
2. a validation phase, intended to perform a set of simulations on data obtained from the literature or identified directly on RCC sections of obese patients.

The approach adopted in the first phase is detailed in the Results section. The tests performed in the second phase, although served mainly as validation for the previous one, also represents the starting point for further studies based on experimental data.

#### Obtaining the Cellular Densities for the Second Phase

The cellular densities chosen for conducting the second phase of our study are summarized in **Table 5**; the majority of the values has been derived from the literature.

Specifically, according to the data published by Gedye et al. (2016), the total number of RCC tumor cells per *mm*^3^ has a minimum value of 2.5 × 10^4^. The median number of CD8+ T cells varies among studies; we used as reference the value obtained by Li et al. (2020) of 84 cells per *mm*^2^. To obtain the density of the other immune cells, we used the immune cell type absolute fraction data for ccRCC from the Cancer Immunome Atlas (TCIA) database (data available at https://tcia.at/cellTypeFractions).

We inferred the number of immune cells per *mm*^3^ by applying the formula proposed by Erdag et al. (2012), that is:

$$cells/mm^{3}=number\;cells/mm^{2}\times \left(1/cell\;diameter\left(in\;mm\right)\right).$$

Then, we calculated the corresponding values in *mL* (or *cm*^3^). In this way, we obtained all the cell densities for lean subjects reported in **Table 5**, except for the mast cells value.

We derived the density of this cell type in lean and obese patients directly by analyzing a RCC section. We also adopted this approach to calculate the cell density ratio between lean and obese patients for the pDCs and NK cells, since they are reported to be significantly different in the two cases, according to Santoni et al. (2019).

In details, the RCC section of an obese patient were stained with CD56 antibody for NK cells, CD1a for DCs, and CD117 for mast cells. Positive cells were counted in 5 consecutive non-overlapping high-power fields (HPF) 400x magnification (0.237 *mm*^2^/*field*), using a Leitz microscope. The results were expressed as the mean of positive *cells*/*mm*^2^ out of 5 regions of interest.

The ratio calculated from this observations gave a density of 21% higher in pDCs and 33% lower in NK cells for obese subjects, compared to the lean ones. For the remaining cells, we inferred from the data provided in Sanchez et al. (2019), a mean increase of the total number of immune cells of 25%.

## 4. Results

The repast runtime provides an interface to perform batch runs and parameter sweeps. Batch runs make possible to run the model multiple times automatically, while parameter sweeps let the system be run using all the possible combinations of parameters. The simulation outputs are combined in a single CSV file that can be used in a worksheet software or in any programming language.

The first phase of our studies (*verification phase*) has been based on 101 simulation runs using the test cellular densities provided in Table 4. The default random seed was set to be different for each run and between obese and non-obese case simulations. At first, this choice might seem unfair, but we should consider that the patients participating in the real life studies have different genetics, age, lifestyle, etc., and the usage of different seeds to initialize the RNG abstracts the innate differences that exist between those people. If the obesity paradox can be detected by the simulation, then the difference would be seen on average, whatever the random seed is, just like this paradox was observed *in vivo* from different patients (Sanchez et al., 2019).

**Table 4:** Initial cell density values in lean patients used for the simulations of the first phase carried out in our studies.

**Cell type**	**Cellular density** (****×*mL***)**	**Cellular density (**×10^−4^*mL***)**
Cytotoxic T Cells	80,000	8
Neutrophils	40,000	4
Mast Cells	40,000	4
Regulatory T Cells	40,000	4
Plasmacytoid Dendritic Cells	40,000	4
Dendritic Cells	80,000	4
M1 Macrophages	40,000	4
M2 Macrophages	40,000	4
Natural Killer Cells	160,000	16
CD4+ Navie T Cells	80,000	8
CD8+ Navie T Cells	80,000	8
Helper Type 1 Cells	40,000	4
Helper Type 2 Cells	40,000	4

*The second column shows the values scaled proportionally to the size of the simulation volume (10^−4^ mL in this phase). In obese individuals, 50 extra agents of the T family, dendritic cell or M1 macrophage type are added picked at random, for a 50% increase of the total number of agents; alongside the number of NK cells is reduced of 16,000 units, with a corresponding increase in pDCs and mast cells*.

In our studies, the duplication rate for tumor cells was a reasonable value considering that it takes 15 steps for the immune system to react since the appearance of the first tumor cell. The difference in proportions of natural killer cell agents compared to mast cell agents and pDC agent was set to 20, so an obese individual had 0 NK cells and 15 of each of the other two, while a lean individual had 20 NK cells and 5 of each of the other two. The Obese M2 Macrophage Mutation Effect, Obese NK Cells Kill Rate Effect, and Obese Treg Differentiation Effect parameters correspond to the effects that are globally enabled in the environment of simulations regarding obese individuals, and have been arbitrarily set to 5 to have a rather strong effect (relative to the probabilities they are going to affect). The Obese M2 Macrophage Mutation Effect parameter also sets the same value but with negative sign for the M1 Macrophage Mutation Effect, which is too globally enabled in the environment. Lastly, the Perinephric Fat Immune Cells parameter is set to 50, so 50 extra agents of the T family, DC or M1 macrophage type are added picked at random, for a 50% increase of the total number of agents in obese individuals.

**Figure 7:**
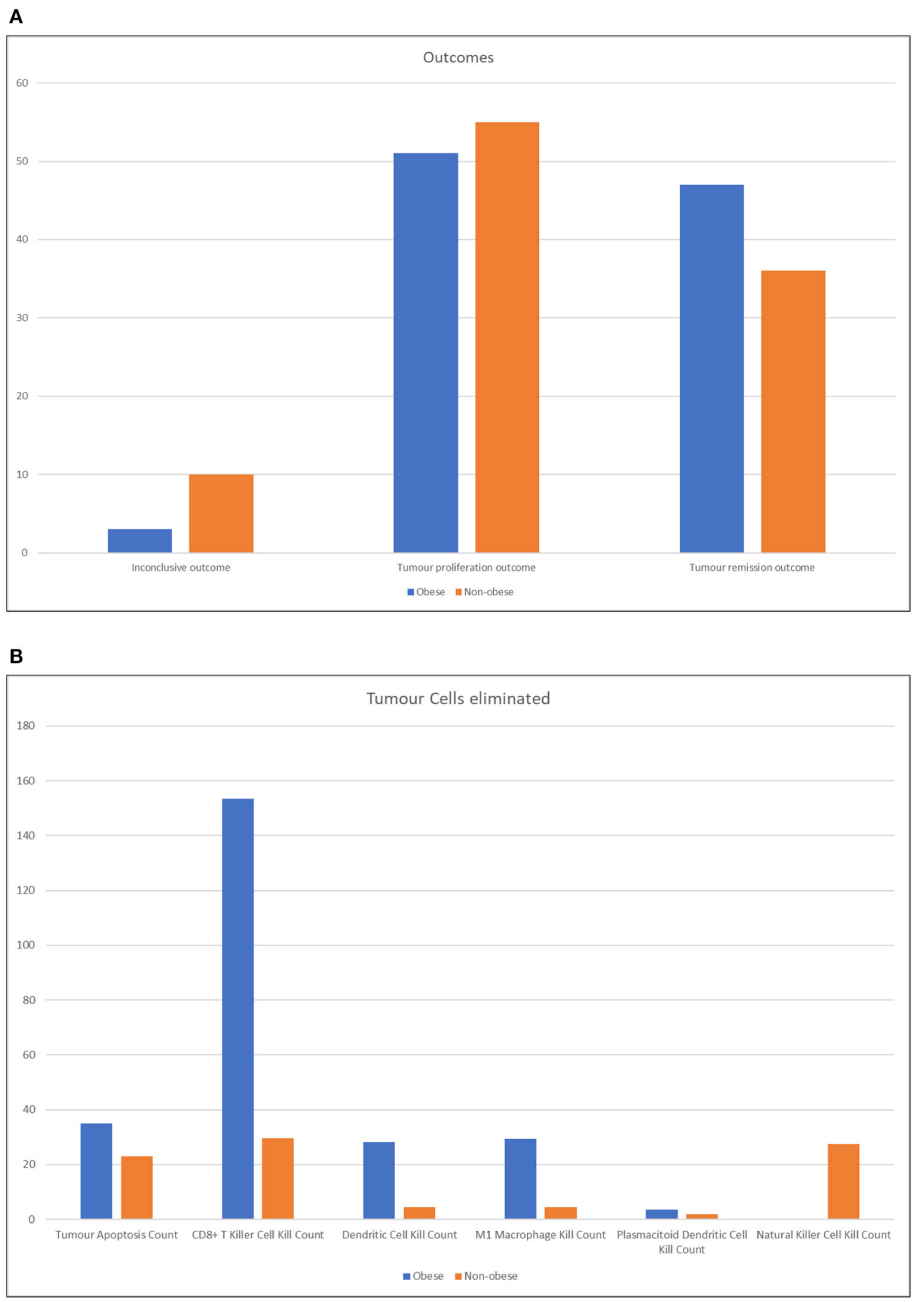
Column charts representing the output of batch simulation from two different perspective. In **(A)**, the outcomes of the simulation are shown, tallied by type. Obese patients had more RCC remission (positive) outcomes and less inconclusive or wholly negative outcomes. See section 3.2 for more details on the outcomes of the simulation. The chart **(B)** represents the RCC cells eliminated by agent type, averaged across runs. Lean patients rely more on natural killer cell agents and secondarily on CD8+ cytotoxic T cells; conversely, the latter have a higher impact on the immune reaction in obese patients.

By observing the outcomes of the batch simulations (see Figure 7A), we notice that obese patients had more RCC remission (positive) outcomes and less inconclusive or wholly negative outcomes. As described in section 10, a *tumor remission outcome* means that the RCC cell agents have been completely eliminated, and consequently that the immune system was better equipped to fight it and the patient would have better prognosis; conversely, an *inconclusive outcome* is obtained when the immune system has somewhat been able to slow down the tumor exponential expansion and prevent its excessive proliferation (i.e., the negative outcome).

It is possible to interpret these results by conjecturing that the RCC microenvironment of obese patients has a strong tendency to converge to one of the more definitive outcomes, while normal patients have a faint tendency for more nuanced outcomes. Despite this, on average, it still means that obese patients have an edge in expected lifetime.

As shown in Figure 7B, the number of RCC cell agents eliminated by agent type or by apoptosis cell death, which can, for example, be triggered by CD4+ Helper 1 T-cell agents effects, averaged across all runs. As expected, non-obese patients rely more on NK cell agents and only secondarily on CD8+ cytotoxic T cells. Obese patients, instead, have a very strong tendency to depend on the action of CD8+ cytotoxic T-cell agents. The very high number of average kills by these agents is due to the fact that while lean patients either eliminate the tumor before it grows too strong or completely lose control quickly, obese patients' immune systems tend to fight longer and destroy a very high number of cells before either a positive or negative outcome arises.

**Figure 8:**
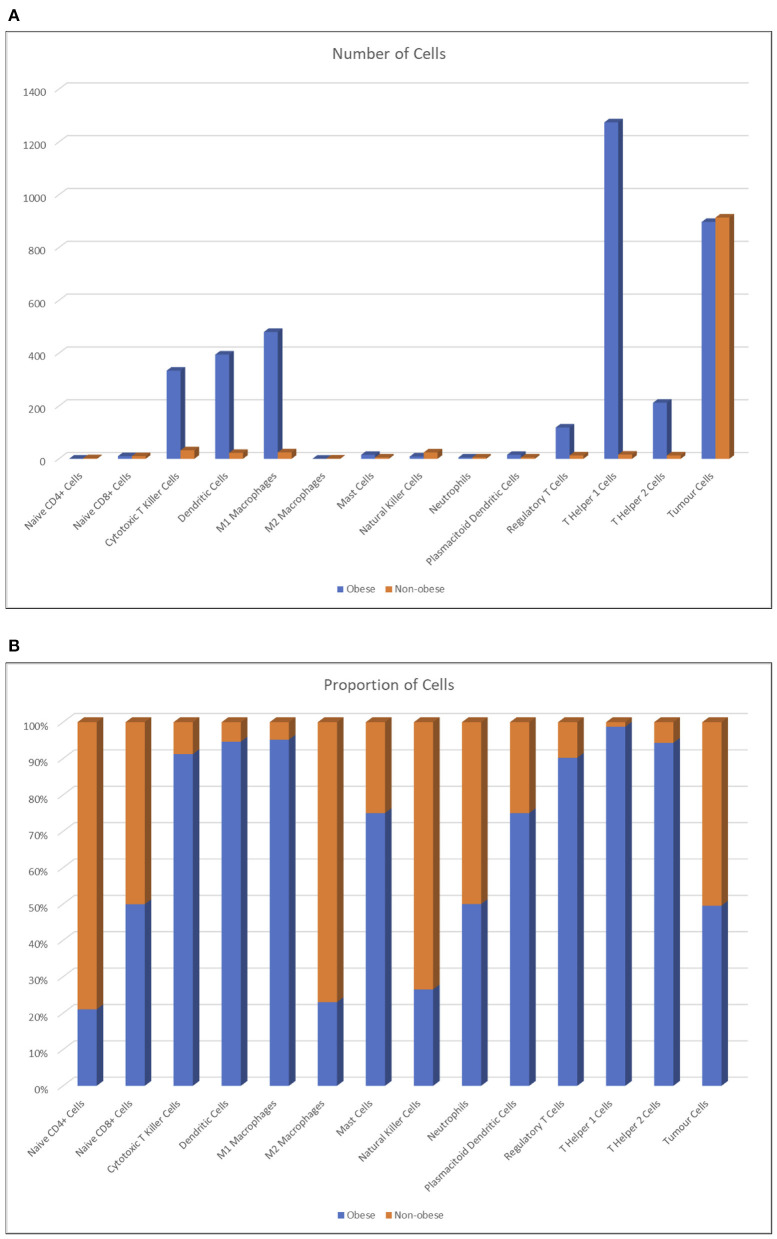
Number of agents averaged across runs **(A)** and percentage proportion of agents between lean and obese individuals, averaged across runs **(B)**.

By comparing the number of agents present at the end of each simulation (Figure 8), the huge number of CD4+ helper 1 T cell agents is evident in obese individuals compared to non-obese patients; we suppose that this result is due to the mutual feedback loop with M1 macrophages. In general, higher numbers of the cells found to be more abundant in the renal fat tissue are reflected in the big spikes in Figure 8A. Prospectively (Figure 8B), lean individuals are better represented by NK cells, but they have also more M2 macrophages and CD4+ naive T cells, which neither differentiate into regulatory T cells nor become activated. The very high numbers reported in obese individuals are reflected only in a very limited increase of positive outcomes seen in Figure 8A, but this is due to the fact that most of these agents do not get to interact much with the tumor, and also that the most crucial steps are those at the early stages of the simulation; once the tumor has grown too much, no amount of immune system cells can stop the exponential growth, even when destroying so many RCC cells as seen in Figure 8B.

We validate the results described above in the second phase of our studies by using the experimental data reported in Tables 5, 6; we obtained this data from the literature and directly by analyzing RCC sections of obese patients (see section 3 for further details). Moreover, in this phase, the tumor is already present at the beginning of the simulation, which means that the simulations represent immediately the immunotherapy treatment.

**Table 5:** Initial cellular densities, in lean and obese patients, used during the validation phase of our studies.

**Cell type**	**Lean** (****×*mL***)**	**Obese (mL)**	**Lean** (****×10^−5^*mL***)**	**Obese** (****×10^−5^*mL***)**
Cytotoxic T Cells	6,000,000	7,500,000	60	75
Neutrophils	21,716,150	21,716,150	217	217
Mast Cells	266,667	600,000	2	6
Regulatory T Cells	142,800	178,500	1	1
Plasmacytoid Dendritic Cells	1,173,850	1,190,003	11	11
Dendritic Cells	1,173,850	1,467,312	11	14
M1 Macrophages	4,360,000	5,450,000	43	54
M2 Macrophages	6,540,000	6,540,000	65	65
Natural Killer Cells	3,271,430	1,090,480	32	10
CD4+ Navie T Cells	4,905,000	4,905,000	49	49
CD8+ Navie T Cells	6,000,000	6,000,000	60	60
Helper Type 1 Cells	2,452,500	2,452,500	24	24
Helper Type 2 Cells	2,452,500	3,065,625	24	30

*The values in the first and second columns are needed to determine the actual number of a specific immune cell type at the beginning of the simulation (shown in the third and fourth columns), according to the chosen simulation volume (10^−5^ mL in this phase). See the Methods section for further details on the approaches used to obtain the provided values*.

**Table 6:** Parameters for the simulations performed in the validation phase of our studies.

**Parameter**	**Value range**	**Description**
Body Mass Index	from 18.5 to 40.0	Value used to determine a lean or obese individual, with cutoff point at 30
First RCC Cell Distance From Blood Vessel	50 μ*m* (Baish et al., 2011)	Distance (in μ*m*) between the blood vessel and the first tumor cell.
RCC growth rate	0.80 *cm* × *year*, from *min* 0.16 to *max* 3.80 (Li et al., 2012)	Number of new cells generated in the time unit.
Perinephric Fat Immune Cells	+ 25%	Percentage increase of the number of immune cells in obese patients, excluding pDCs, mast cells, and NK cells (for which the number is calculated directly).
Volume (*mL*)	10^−5^	Value used to compute the dimension of the simulation grid.

*They are used along with the experimental values for cellular densities reported in Table 2*.

**Figure 9:**
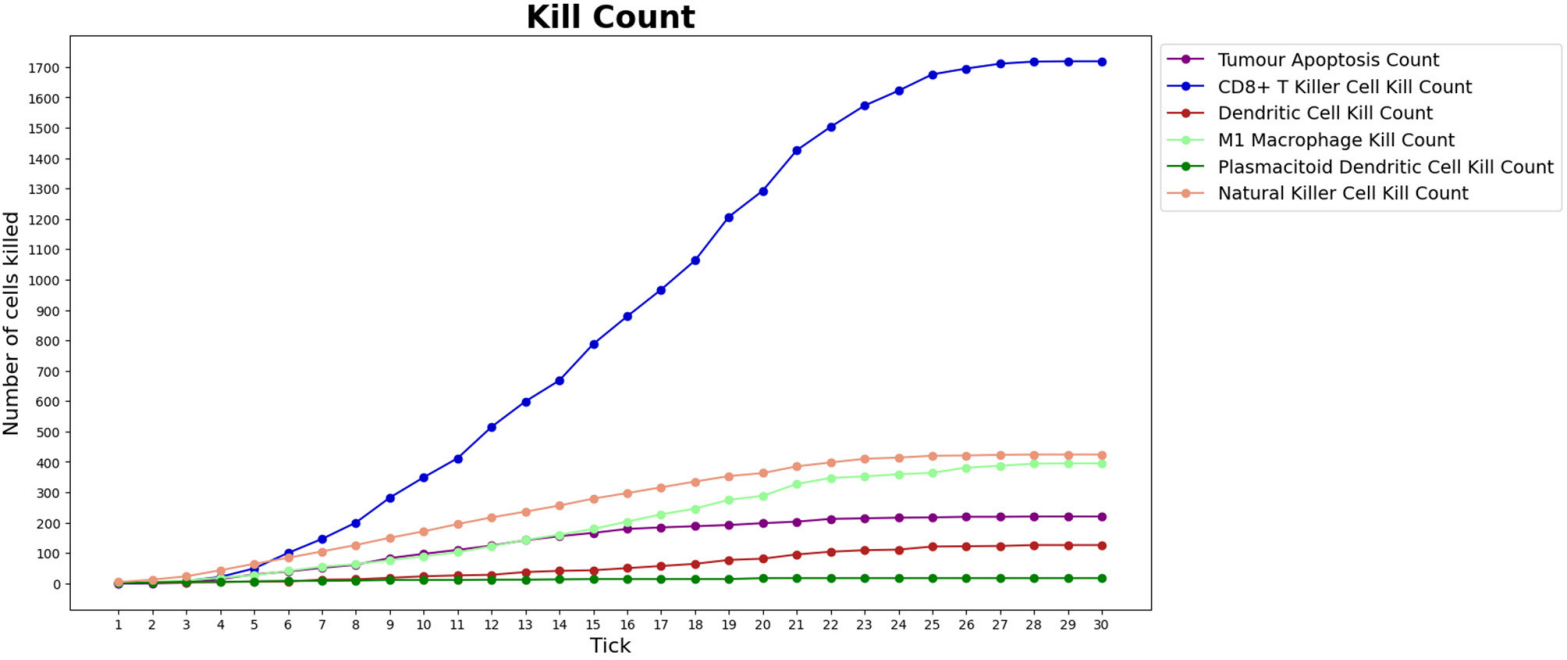
Number of tumor cells eliminated by each type of BioAgent, in a lean patient, during a simulation performed as part of the validation phase of our work. Compared to Figure 6, we can observe a similar behavior of the immune cells, in particular regarding the higher killing activity of CD8+ T killer cells and NK cells compared to the other cell types. In the implementation of the validation phase, the tumor is already present at the beginning of the simulation and the immunotherapy starts immediately, meaning that we do not need to wait for the tick 15 to see the immune cells attacking the tumor.

Although this phase is still at its first stages, the result we obtained so far are already in favor of the outcome of the previous phase. Indeed, as shown in Figure 9, for lean patients, we observed similar killing rates compared to those resulted from the first phase.

## 5. Discussion

In this article, we provide an agent-based model of the RCC microenvironment in order to investigate the obesity paradox. The environment is described as a 3D space with a cubical shape in which the agents interact influenced by the BMI parameter and by the presence of a blood vessel.

The system is stochastic, that is, it relies on a random number generator for the agents' action, placement, and, in some cases, behavior. The pathways and proteins involved in real RCC microenvironments have been modeled as effects that influence the stochastic decisions and constrain agents.

Various types of immune cells have been modeled on the basis of their observed behavior in the literature and are described in detail. The model can be run within the repast runtime and provides an interface for the most relevant parameters. During the simulation the evolution of the system can be visualized using 3D projections and, additionally, through time series that show the number of agents and RCC cells eliminated by type.

By running the model in batch, it was discovered a limited improvement in the outcomes of obese individuals. The model was shown to accurately depict the differences in the environments of lean and obese individuals, the former having most of the work carried out by NK cells and the latter shifting the burden on CD8+ cytotoxic T cells. The correct proportions of cells was also maintained during the simulations.

We started to validating our simulation approach basing on experimental data, obtaining promising results. However, this phase will proceed in the near future since it needs more experimental data and computational time.

Our model can be further expanded. Indeed, we might improve its faithfulness to the pathways and protein signaling of living cells by adding new cell agent types and effects.

Moreover, to better support the design of new immunotherapy treatments, we can introduce more parameters, representing the properties of the tumor microenvironment we do not take into account in the present model. As an example, we did not model cell lifetimes, but we may implement this property by removing a cell agent from the environment after a time interval, defined through a newly defined parameter. As a further work, we aim to construct a topological classifier suitable to distinguish different microenvironments and identify the class of *reversible RCC behaviors* (Piangerelli et al., 2018).

Our idea is to define a model of a system whose behavior is function of the fat concentration in the environment; therefore, it does not take into account the behavior of the adipocytes, but only their quantity. In the literature, the study of the spatiotemporal dynamics of the tumor immune response in the context of the immunotherapy treatment have been already modeled through quantitative agent-based approaches (Gong et al., 2017). However, as suggested by Norton et al. (2019), drug delivery and response processes are mostly qualitative, a limitation that might be overcome by integrating the agent-based approach with physiologically based pharmacokinetic and pharmacodynamic models (Cosgrove et al., 2015). In future works, we might apply this idea to our models to simulate the effects of tumor growth and angiogenesis inhibitors for the treatment of the RCC, such as pazopanib and sunitinib (Santoni et al., 2014, 2015).

The aim of this research is to build a computational framework to support oncologists during the immunotherapy design and monitoring in patients affected by RCC. The computational model underlying the framework will be built with a learning process that exploits open access data and data collections owned by the institution where the physician operates. The learning process is based on a tumor model dynamically defined during the monitoring of the immunotherapy treatment (delivered to different patients), which, once simulated in the computational framework, will reveal the characteristics emerging in the personalized model of the patient that will assist the oncologist to predict the value of the immunotherapy drug.

## Data Availability Statement

The original contributions presented in the study are included in the article/Supplementary Material, further inquiries can be directed to the corresponding author/s.

## Author Contributions

EM designed the research. MB and GR implemented the method. MB, GR, SM, AC, RM, MS, and EM performed the research. MS, AC, and SM analyzed the data. MB and SM wrote and EM revised the paper. All authors contributed to the article and approved the submitted version.

## Conflict of Interest

The authors declare that the research was conducted in the absence of any commercial or financial relationships that could be construed as a potential conflict of interest.

## Footnotes

^1^According to the WHO, a patient is obese if the BMI is at least 30 *kg*/*m*^2^; a BMI is the body mass index defined as the person's weight in kilogram divided by the square of person's height in meter. The BMI of a normal patient is between 18.5 and 24.9 *kg*/*m*^2^.
