# Supplementary material for: Agent-Based Learning Model for the Obesity Paradox in RCC
Source: Front Bioeng Biotechnol. 2021 Apr 29;9:642760. doi: 10.3389/fbioe.2021.642760 (PMC8116955; doi:10.3389/fbioe.2021.642760)
Supplement: Supplementary file 1 [file Data_Sheet_1.pdf]

Supplementary Information to
Agent-based Framework for Learning the Obesity Paradox in RCC

1 IMMUNE CELL BIOAGENTS

We model the tumour microenvironment as a collection of agents (we also refer to them as BioAgents); basing on this approach, we developed a multiagent simulator called OncoAgent[1].

In this section, we show the type of actions that each agent can perform and the effects it can have on other agents (see Section 3.1 of the main article for more details on the effects that an agent can generate or being subjected to). Oftentimes, a probability is associated with a certain action or effect and specifies whether the action succeeds or if the effect is generated. In such cases, we do not provide the exact probability, because it is arbitrarily defined, but we report the events by which it can be conditioned. Likewise, we outline the relative strength of an effect compared to another. Using arbitrary probabilities does not affect the validity of the simulation, as long as they are consistent in both the cases of a lean and an obese subject simulation; since the aim of our in silico study is to evaluate whether obese patients have better prognoses compared to their lean counterparts, this probabilistic approach results to be a viable approximation (in Section 2, we will provide more information on the choices made to determine the probability values).

Among the cell types that compose the immune system, we identified and modelled six categories of interest for our studies; for three of them, we also took into account the behaviour of specific cell subpopulations.

The BioAgent types defined in our model are summarised in the following Table S1.

**BioAgent**	**Behaviour**
T cells	CD4+ naive<br>CD4+ helper 1<br>CD4+ helper 2<br>CD8+ naive<br>CD8+ cytotoxic<br>Regulatory (Treg)
Dendritic cells	Conventional (cDC)<br>Plasmacytoid (pDC)
Macrophages	M1 Phenotype<br>M2 Phenotype
Mast cells	Mast cell
Natural killers	Natural killer
Neutrophils	Neutrophil

Table S1. Overview of the BiaAgent categories, and the related behaviours, that we derived from the immune cell types taken into account in our models.

[1] https://bioshape-and-data-science-lab.github.io/OncoAgent

In the rest of this section, we will describe the behaviours of all them. For each BioAgent type, we also provide a brief introduction of the functions carried out by its biological counterpart in a living system.

1.1 T Cell Agents

1.1.1 CD4+ Naive T Cell

The CD4+ naive T cell represents the naive form of T helper cells. A naive T cell is considered immature and, unlike the activated or the memory T cells, has not come into contact with its cognate antigen. Naive T cells can respond to novel pathogens, which the immune system has not yet encountered. If activated, they differentiate into CD4+ helper 1 T cell or CD4+ helper 2 T cell. Recognition by a naive T cell of its cognate antigen results in the initiation of an immune response.

CD4+ Naive T Cell agents have an associated an 8 bit string that represents their antigen receptors. The agent does not do anything until an APC (such as a conventional dendritic cell, a plasmacytoid dendritic cell or a M1 macrophage) attempts to activate it. When this event happens, the Hamming distance between the agent's bit string and the presented antigen (in this case, the tumour's) bit string is calculated, and if it is below a specified threshold (by default 3), the activation is considered to be successful. This is the same mechanism at the core of the Celada-Seiden model for antigen recognition (Celada and Seiden, 1992). In case of activation, the agent differentiate into either CD4+ helper 1 T cell or CD4+ helper 2 T cell, with equal probability. If the activation fails, the agent is removed and then recreated in a different location with a new bit string. Additionally, there is a chance for the agent to differentiate into a regulatory T cell, and this event is influenced by the `Regulatory T Differentiation Effect`. In reality, CD4+ Naive T Cells do not become Regulatory T cells (Treg), but since Tregs share the same phenotype (i.e. they, too, present the CD4+ biomarker), their naive form was modelled using the same agent.

1.1.2 CD4+ Helper Type 1 Cell

The T helper cells play a relevant role in the adaptive immune system by helping the regulation of immune responses. In particular, the T helper type 1 lineage carries out an anti-tumour activity, which involves the activation of dendritic cells (Mailliard et al., 2002) and of the M1 macrophages; they also directly kill the tumour cells through the TNF-related apoptosis-inducing ligand (TRAIL) pathway (Lin and Karin, 2007).

CD4+ Helper Type 1 Cell agents perceive and move towards the tumour mass and stay there. They have a chance to activate new dendritic cell and M1 macrophage agents that appear at the edge of the environment. They additionally have a chance to proliferate, which is influenced by the `Helper 1 T Proliferation Effect`. This possibility is kept small, to prevent an uncontrolled growth. Lastly, they generate a positive `Tumour Apoptosis Effect`.

1.1.3 CD4+ Helper Type 2 Cell

The other main subtype of helper T cells is represented by the Helper Type 2 cells. Their anti-tumour activity primarily involves recruitment of specific white-blood cells, the eosinophils, to the tumour environment. Anti-tumour eosinophil activity includes attraction of tumour-specific CD8+ cytotoxic T cells and activation of the M1 macrophages (Carretero et al., 2015). Additionally, the presence of helper 2 T cells is associated with tumour proliferation (Kim and Cantor, 2014).

CD4+ Helper Type 2 Cell agents activate new M1 macrophage agents and attract CD8+ cytotoxic T cell agents, which appear at the edge of the environment. Moreover, they have a chance to proliferate, which is kept small to prevent uncontrolled growth. Eosinophils are not directly modelled, but their behaviour

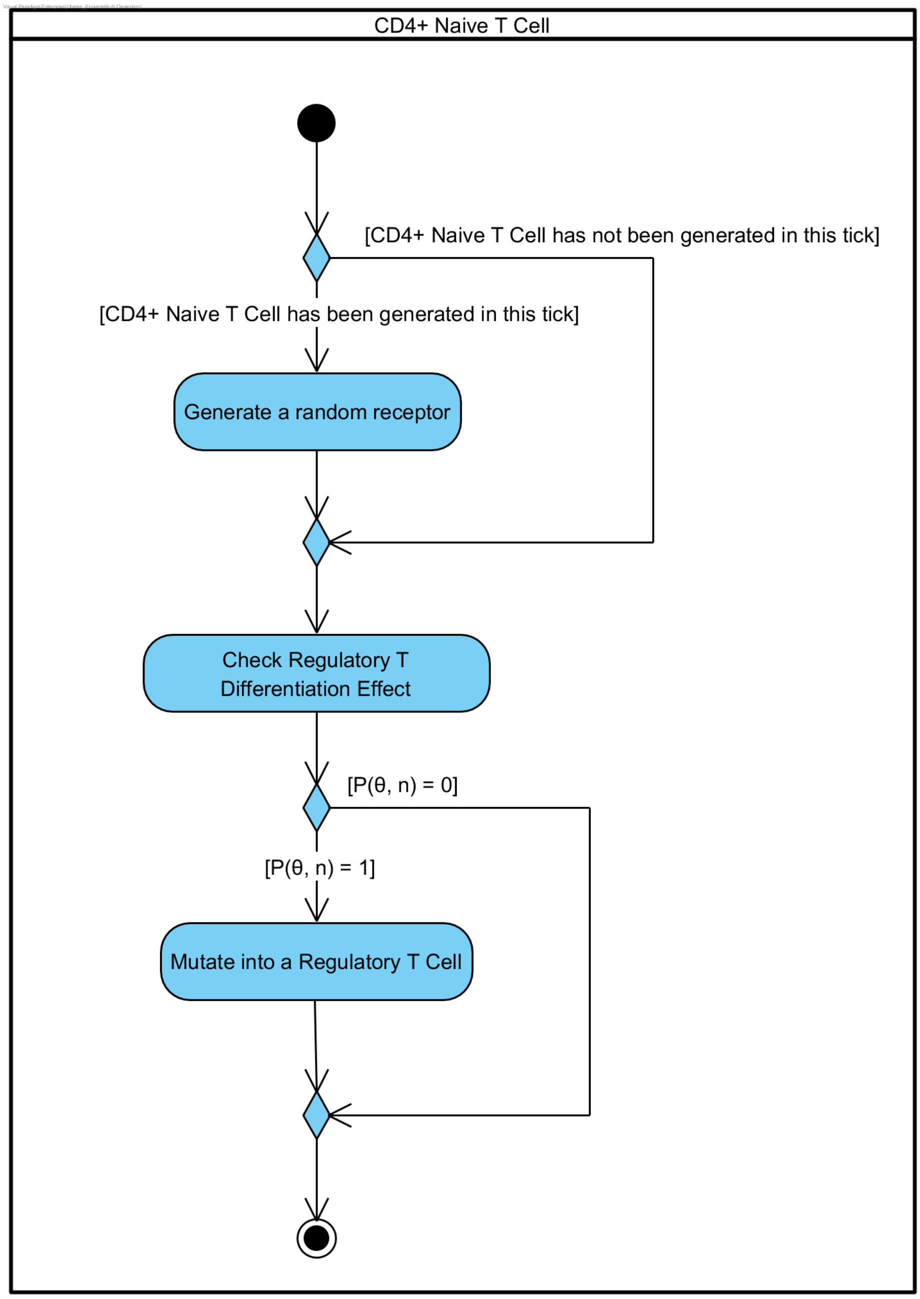

Figure S1: This activity diagram shows the behaviour of a CD4+ Naive T Cell at each time step of the simulation since the start of the treatment. Each CD4+ Naive T Cell has its own randomly generated receptor that can bind to a range of antigens when a Tumour Cell is presented to it by M1 Macrophages, Dendritic Cells or Plasmacitoid Dendritic Cells. The CD4+ Naive T Cell has a chance, influenced by any present Regulatory T Differentiation Effect, to mutate into a Regulatory T Cell.

has been represented as effects produced by the CD4+ Helper Type 2 cell agents; they generate a positive `Cytotoxic T Cell Kill Rate Effect` and a positive `Tumour Growth Effect`.

1.1.4 CD8+ Naive T Cell

The CD8+ naive T cell represents the naive form of CD8+ cytotoxic T cells. They can react to novel pathogens but need to be activated by an antigen-presenting cell (APC) to mount an immune response.

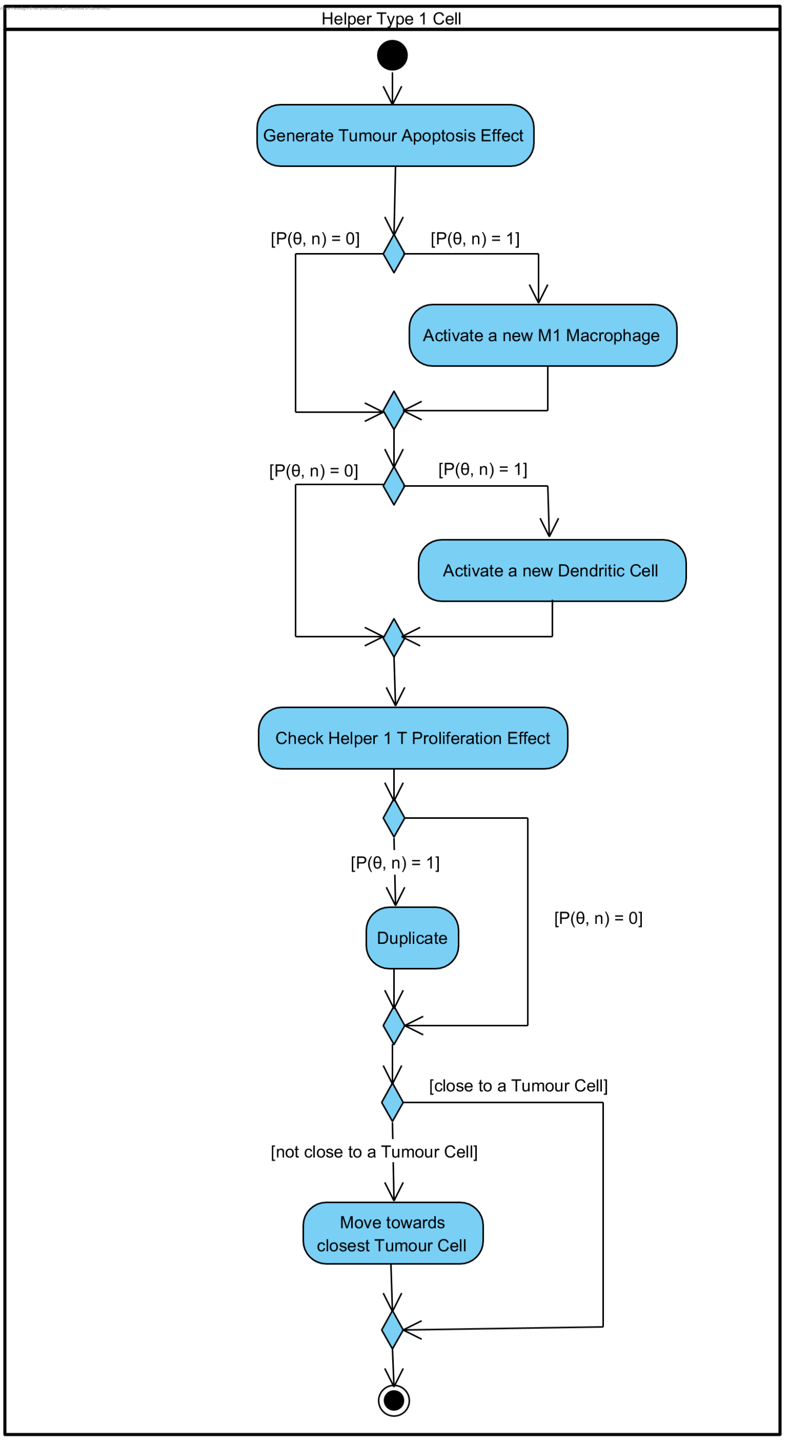

Figure S2: This activity diagram shows the behaviour of a Helper Type 1 Cell at each time step of the simulation since the start of the treatment. The Helper Type 1 Cell exposes only a Tumour Apoptosis Effect. It has a chance to activate a new M1 Macrophage or a new Dendritic Cell and, after taking into account the effect of any present Helper 1 T Proliferation Effect, it may duplicate and then move towards the closest Tumour Cell.

CD8+ Naive T Cell agents, like their CD4+ counterpart, have an 8 bit string, different (but there can be repetitions) for each agent instance. The agent does not do anything until activated by an antigen-presenting

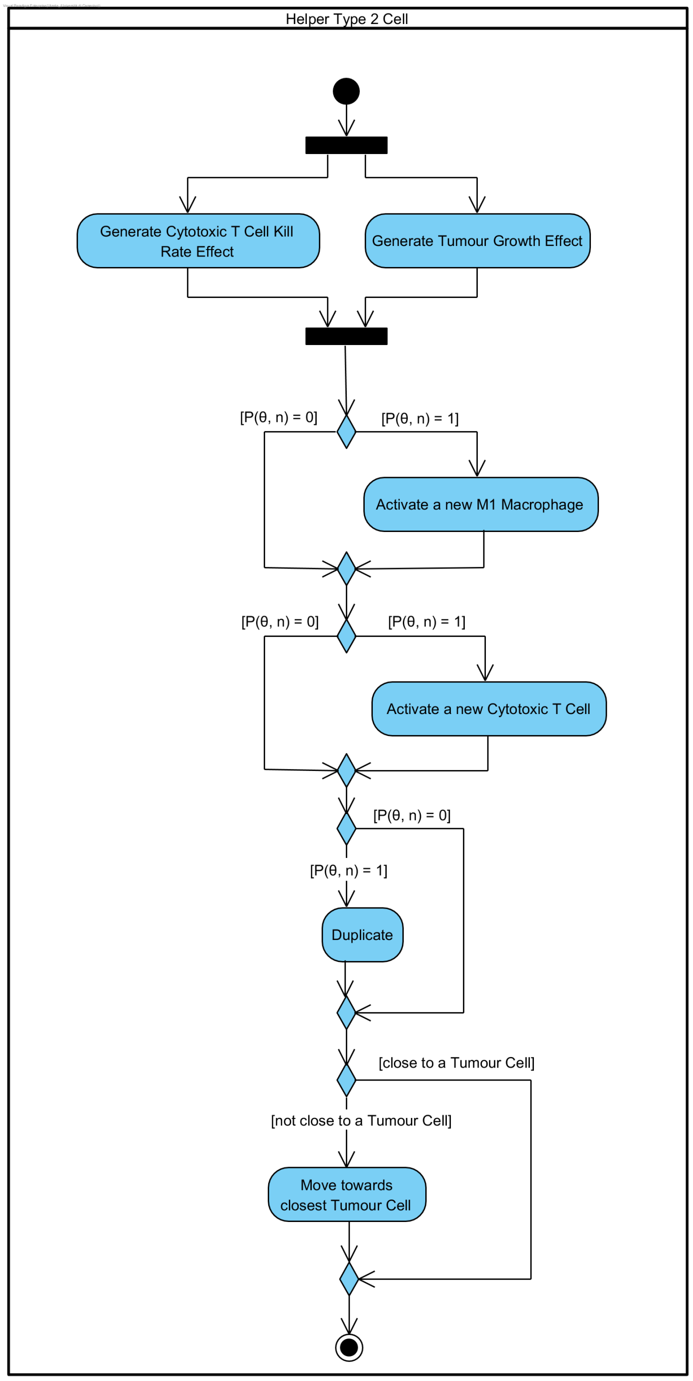

Figure S3: This activity diagram shows the behaviour of a Helper Type 2 Cell at each time step of the simulation since the start of the treatment. The Helper Type 2 Cell exposes Cytotoxic T Cell Kill Rate Effect and a Tumour Growth Effect. It has a chance to activate a new M1 Macrophage or a new Cytotoxic T Cell, it may duplicate and it will move towards the closest Tumour Cell.

cell (APC). In this case, though, the `Cytotoxic T Cell Activation Effect` can influence the threshold that the Hamming distance between the agent's and the tumour's bit strings must stay within. In case of successful activation, the agent turns into a CD8+ cytotoxic T cell, while in case of failure, the agent is removed and then recreated in a different location with a new bit string. This is done to model the continuous creation of new naive T cells, both in this and the CD4+ naive T cell case.

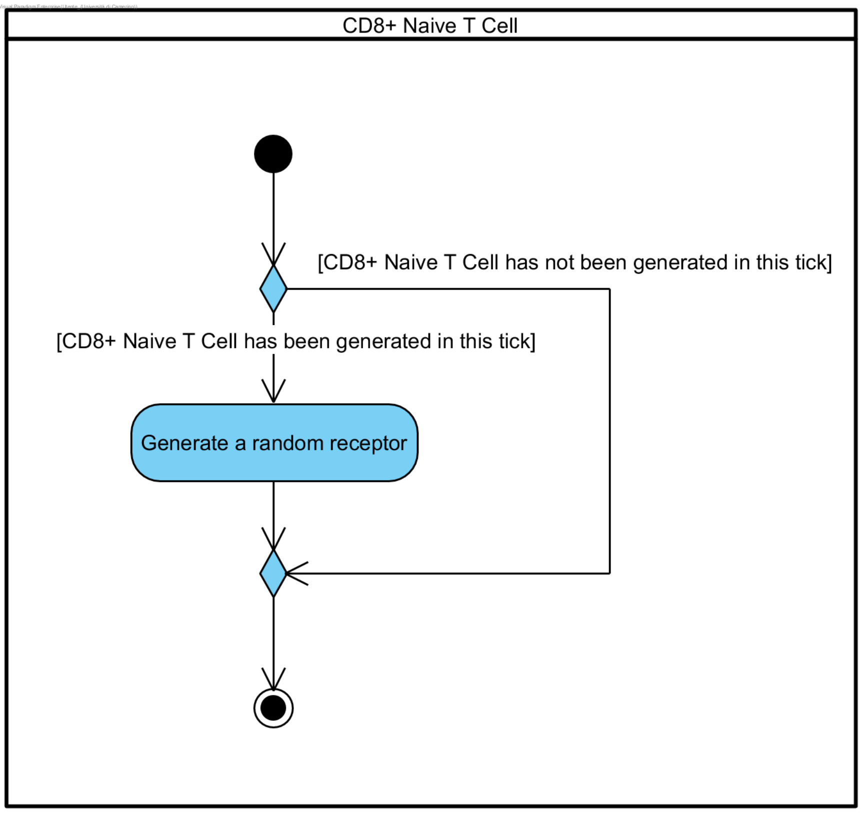

Figure S4: This activity diagram shows the behaviour of a CD8+ Naive T Cell at each time step of the simulation since the start of the treatment. Each CD8+ Naive T Cell has its own randomly generated receptor that can bind to a range of antigens when a Tumour Cell is presented to it by Dendritic Cells or Plasmacitoid Dendritic Cells.

1.1.5 CD8+ Cytotoxic T Cell

CD8+ cytotoxic T cells are a type of white blood cell able to destroy cancer cells and other types of damaged cells.

CD8+ Cytotoxic T Cell agents represent the activated version of the CD8+ naive T cell agent. In this case, it is able to recognise the tumour antigen.

They perceive and move towards RCC tumour cell agents and attempt to destroy them. This action has a high probability of success and it is influenced by the `Cytotoxic T Cell Kill Rate Effect`. Additionally, this agent has a chance to proliferate and this probability is affected by the `Cytotoxic T Cell Proliferation Effect`. The agent might also undergo apoptosis: this happens when it receives a greater than zero `Cytotoxic T Cell Apoptosis Effect`; the greater it is, the more likely is the cell death to happen.

1.1.6 Regulatory T Cell

The regulatory T cells (Treg) are a subpopulation of T cells whose main function is modulating the immune system. They suppress or down-regulate T cells' proliferation. Regarding the effects of Tregs in the RCC micro-environment, Treg cells are involved in tumour development and progression, since they are able to inhibit antitumour immunity (Ohue and Nishikawa, 2019).

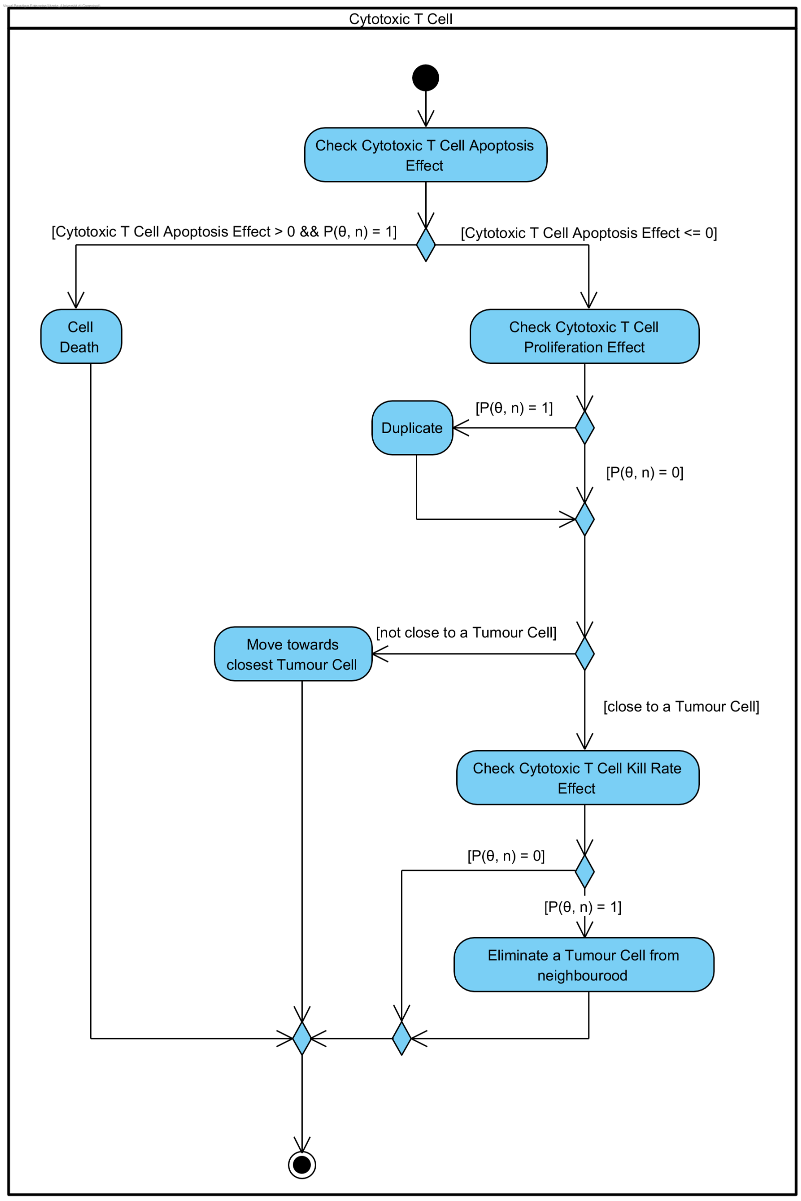

Figure S5: This activity diagram shows the behaviour of a Cytotoxic T Cell at each time step of the simulation since the start of the treatment. The Cytotoxic T Cell might get destroyed as a result of a Cytotoxic T Cell Apoptosis Effect or it may duplicate, this latter action affected by any present Cytotoxic T Cell Proliferation Effect. The Cytotoxic T Cell will move towards the closest Tumour Cell and attempt to destroy it, with the success of this latter action influenced by any present Cytotoxic T Cell Kill Rate Effect.

Regulatory T cell agents have been modelled on the basis of the information provided in Ohue and Nishikawa (2019). They perceive and move towards the tumour mass, while exposing different suppressor effects. This type of agent generates a highly negative `Cytotoxic T Cell Kill Rate Effect` and `Cytotoxic T Cell Proliferation Effect`, positive `Cytotoxic T Cell Apoptosis Effect`, negative `Cytotoxic T Cell Activation Effect` and a negative `Dendritic Cell Phagocytosis Effect`.

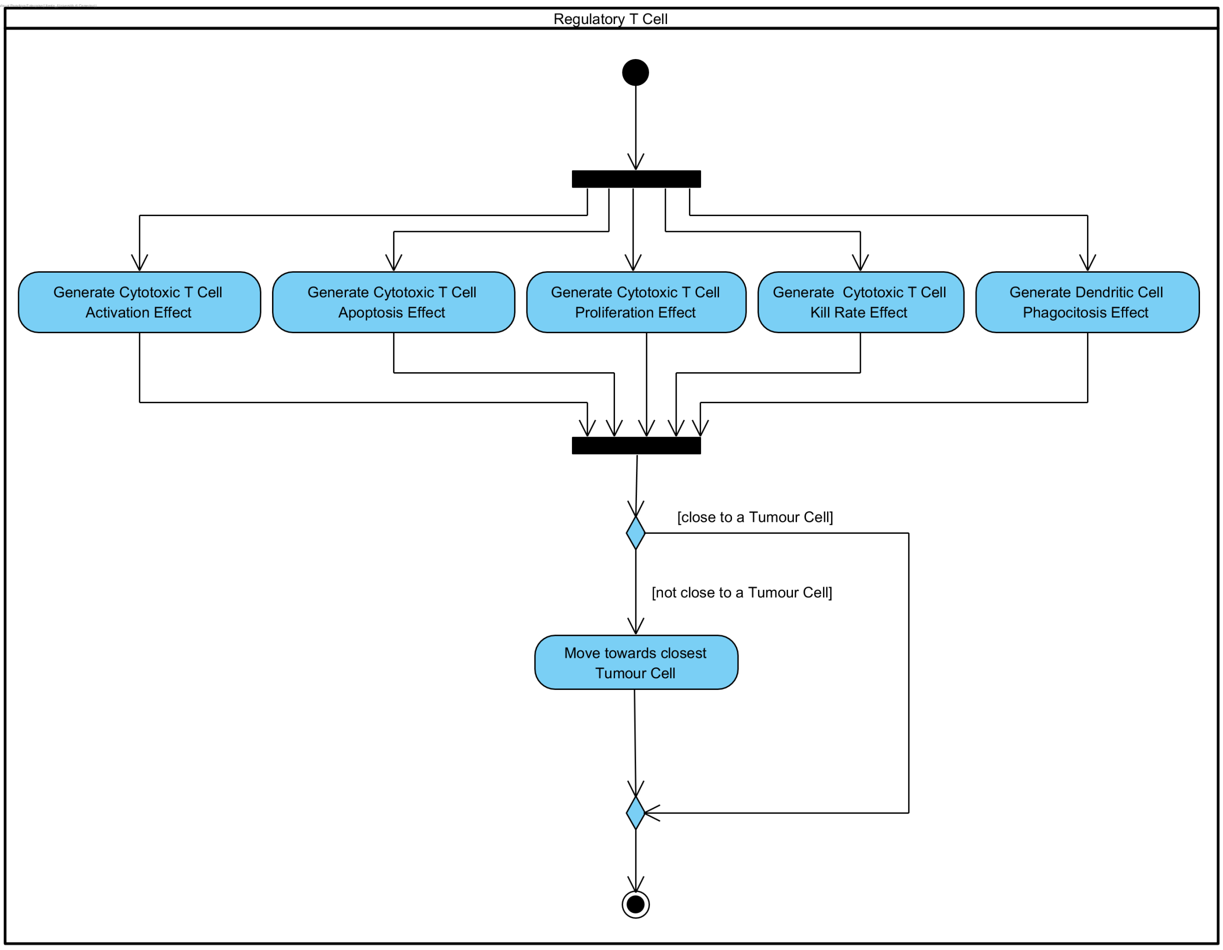

Figure S6: This activity diagram shows the behaviour of a Regulatory T Cell at each time step of the simulation since the start of the treatment. The Regulatory T Cell exposes a Cytotoxic T Cell Activation Effect, Cytotoxic T Cell Apoptosis Effect, Cytotoxic T Cell Proliferation Effect, Cytotoxic T Cell Kill Rate Effect and a Dendritic Cell Phagocytosis Effect. It moves towards the closest Tumour Cell.

1.2 Dendritic Cell Agents

1.2.1 Conventional Dendritic Cell (cDCs)

Dendritic cells are antigen-presenting cells (APCs). Together with M1 macrophages and B cells they are considered professional APCs, specialised in presenting antigens to T cells.

Conventional dendritic cells (cDCs) can present antigens to both CD4+ helper T cells and CD8+ cytotoxic T cells. In particular, they are able to perform cross-presentation and activate CD8+ naive T cells. This behaviour can be observed in the case of Renal Cell Carcinoma (Schwaab et al., 1999).

Conventional dendritic cell (cDC) agents perceive and move towards RCC tumour cell agents and attempt to phagocyte them; this action has a high probability associated and it can be influenced by the `Dendritic Cell Phagocytosis Effect`. If the action succeeds, a RCC tumour cell agent is phagocyted, i.e. removed from the simulation, and the agent can perceive and move towards the closest naive T cell, which can be either CD4+ naive T cell agents or CD8+ naive T cell agents. When it finds one, it attempts to activate it. The activation does not have an associated probability, but it may fail if the T cell does not have the appropriate receptors. In case of success, the phagocyted RCC tumour cell is removed

and the agent goes back to phagocyte more RCC tumour cells. In case of failure, the agent seeks a different T cell.

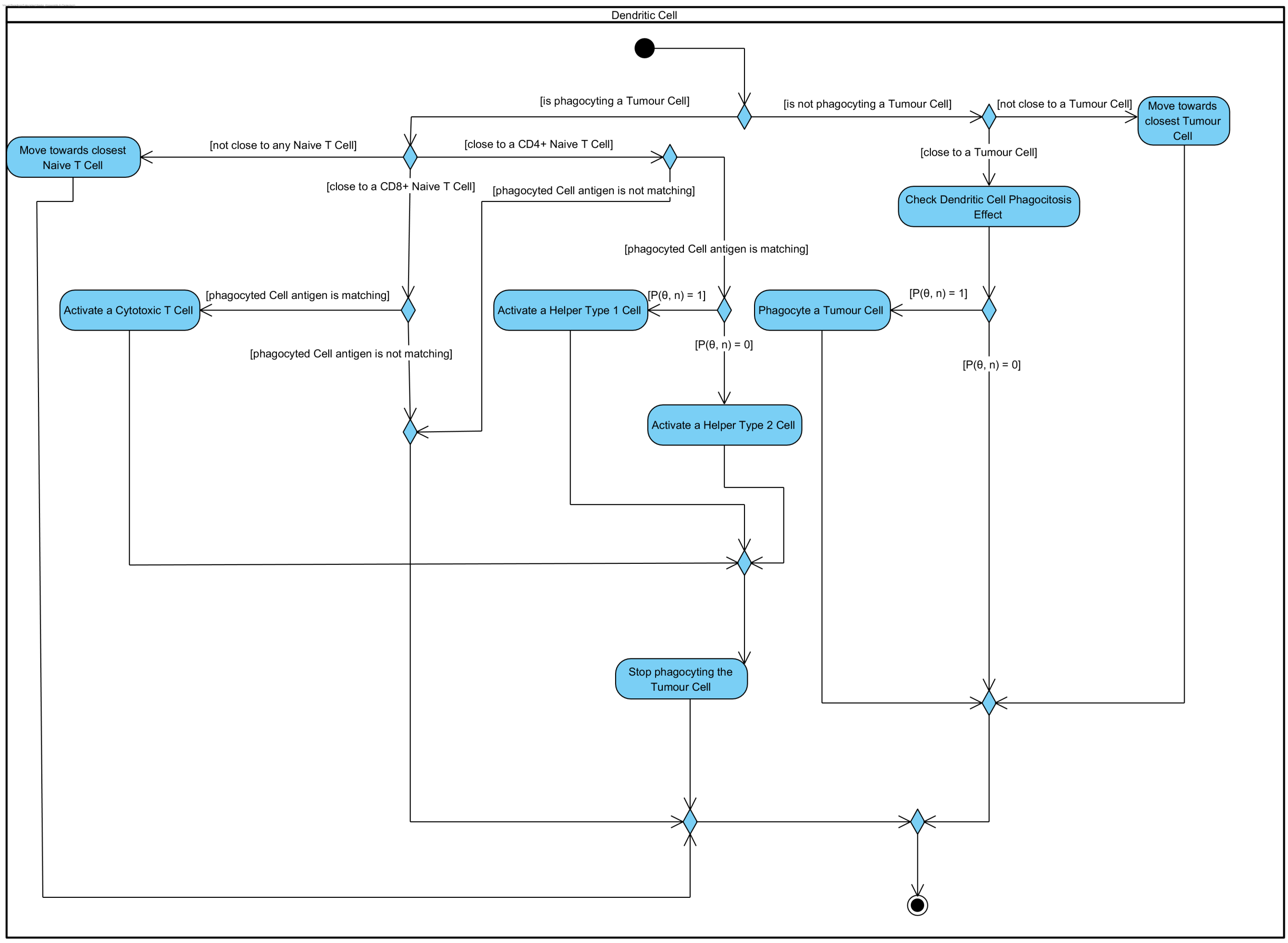

Figure S7: This activity diagram shows the behaviour of a Dendritic Cell at each time step of the simulation since the start of the treatment. The Dendritic Cell will move towards the closest Tumour Cell and attempt to phagocyte it. When phagocyting a Tumour Cell, it can only move towards any available Naive T Cell and attempt to activate it. Activating a CD4+ Naive T Cell will result in the generation of either a Helper Type 1 Cell or a Helper Type 2 Cell. Activating a CD8+ Naive T Cell will result in the generation of a Cytotoxic T Cell. The activation of a Naive T Cell succeeds if there is an appropriate matching between the antigen of the phagocited Tumour Cell and the receptor of the Naive T Cell. After activation of a Naive T Cell, the Dendritic Cell is no longer phagocyting and can therefore attack Tumour Cells again in future time steps.

1.2.2 Plasmacytoid Dendritic Cell (pDCs)

Plasmacytoid dendritic cells (pDCs) play a fundamental role in the immune system, since they are the major producers of type I interferon (IFN type I), an important immune system activity regulator (Koucký et al., 2019).

The behaviour of the *plasmacytoid dendritic cell agents* is similar to that of the conventional dendritic cell agents. In addition, this agents are modelled following the pDC contributions to the tumour promotion and suppression outlined in Koucký et al. (2019). The agents have a chance to activate new natural killer cell agents, which are added into the environment at one of its edges. Moreover they may have any

combination of the following effects: positive `Angiogenesis Effect`, positive `Natural Killer Cell Kill Rate Effect`, positive `Regulatory T Differentiation Effect`, positive `Cytotoxic T Cell Kill Rate Effect`, negative `Cytotoxic T Cell Proliferation Effect`. The subset of these effects that any single plasmacytoid dendritic cell agent may have is chosen randomly (with a 50% chance for each effect to be present) when the agent is created.

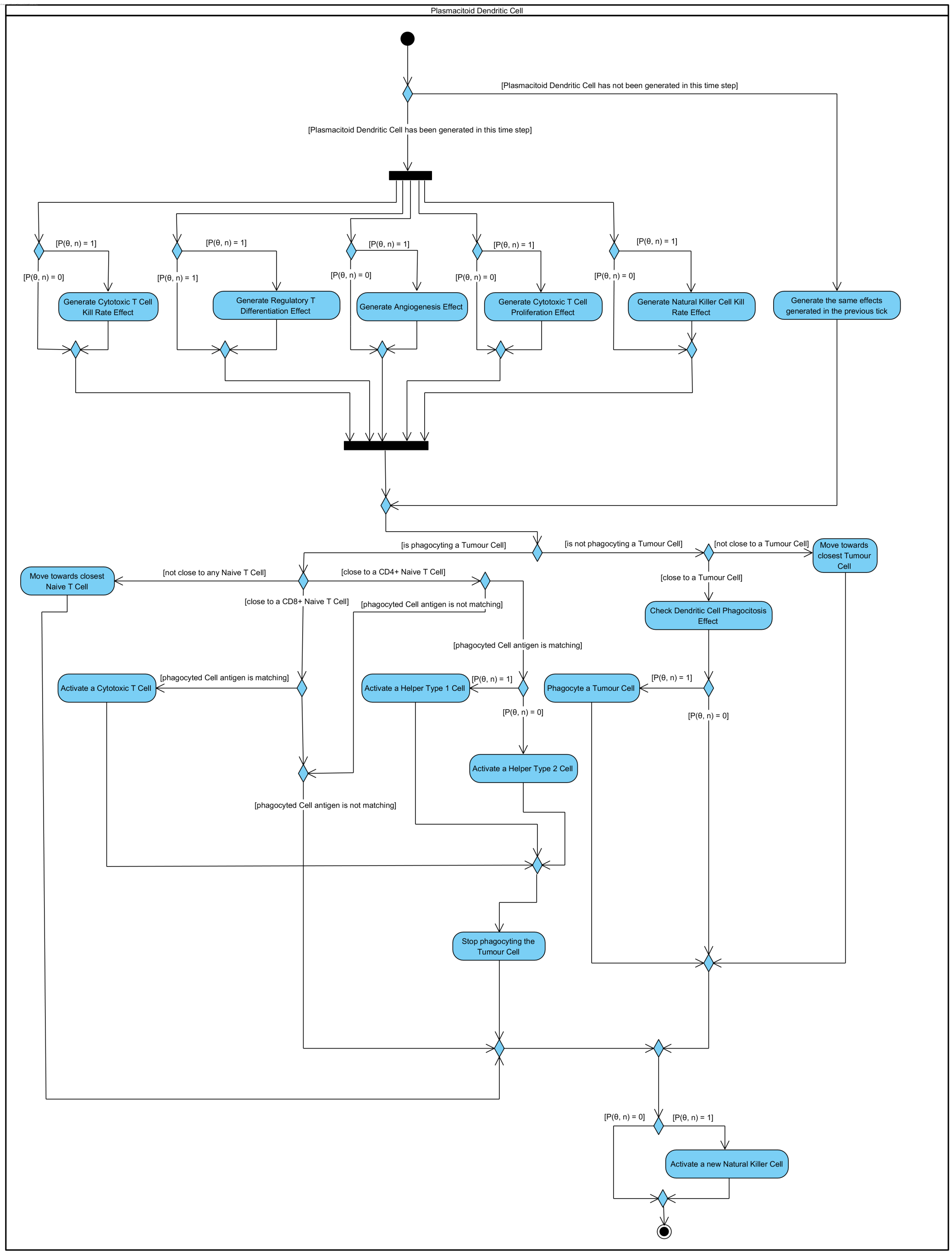

Figure S8: This activity diagram shows the behaviour of a Plasmacitoid Dendritic Cell at each time step of the simulation since the start of the treatment.

The Plasmacitoid Dendritic Cell can expose any combination of the following effects: Cytotoxic T Cell Kill Rate Effect, Regulatory T Differentiation Effect, Angiogenesis Effect and Natural Killer Cell Kill Rate Effect. The pDC will move towards the closest Tumour Cell and attempt to phagocyte it. When phagocyting a Tumour Cell, it can only move towards any available Naive T Cell and attempt to activate it. Activating a CD4+ Naive T Cell will result in the generation of either a Helper Type 1 Cell or a Helper Type 2 Cell. Activating a CD8+ Naive T Cell will result in the generation of a Cytotoxic T Cell. The activation of a Naive T Cell succeeds if there is an appropriate matching between the antigen of the phagocited Tumour Cell and the receptor of the Naive T Cell. After activation of a Naive T Cell, the pDC is no longer phagocyting and can therefore attack Tumour Cells again in future time steps. Furthermore, pDCs have also a chance to activate new Natural Killer Cells.

1.3 Macrophage Cell Agents

1.3.1 M1 Phenotype

Macrophages are a type of white blood cells that ingests (phagocytes) substances and cells external or harmful to the organism, including microbes and cancer cells.

M1 macrophages enhances CD8+ cytotoxic T cells antitumour activity (Vlahopoulos, 2017) and promotes CD4+ helper 1 T cell differentiation (Lin and Karin, 2007).

M1 macrophage agents perceive and move towards RCC tumour cell agents and phagocyte them. Each time a RCC tumour cell is phagocyted, the related agent is removed from the simulation, and the M1 macrophage that completed this action perceives and moves towards the closest CD4+ naive T cell agent. When it finds one, it attempts to activate it. The activation does not have an associated probability, but it may fail if the T cell does not have the appropriate receptors. In case of success, the agent goes back to phagocyte more RCC tumour cells. In case of failure, the agent seeks a different T cell. At every time step, the agent might also undergo a phenotype switch and become a M2 macrophage. This switch happens with low probability but it is influenced by the `M1 Macrophage Mutation Effect`. The agent is also a source of a positive `Cytotoxic T Cell Kill Rate Effect` and a positive `Helper 1 T Proliferation Effect`.

1.3.2 M2 Phenotype

The M2 macrophage phenotype differs from the M1 phenotype in its ability to moderate the inflammatory response and stimulate angiogenesis and tumour growth (Dandekar et al., 2011).

M2 macrophage agents perceive and move towards RCC tumour cell agents and stay as close as possible to the tumour mass. Like their M1 counterpart, at every time step they might switch to the other phenotype with a low probability, in this case influenced by the `M2 Macrophage Mutation Effect`. They generate a positive `Angiogenesis Effect`, a positive `Tumour Growth Effect` and a negative `Cytotoxic T Cell Kill Rate Effect`.

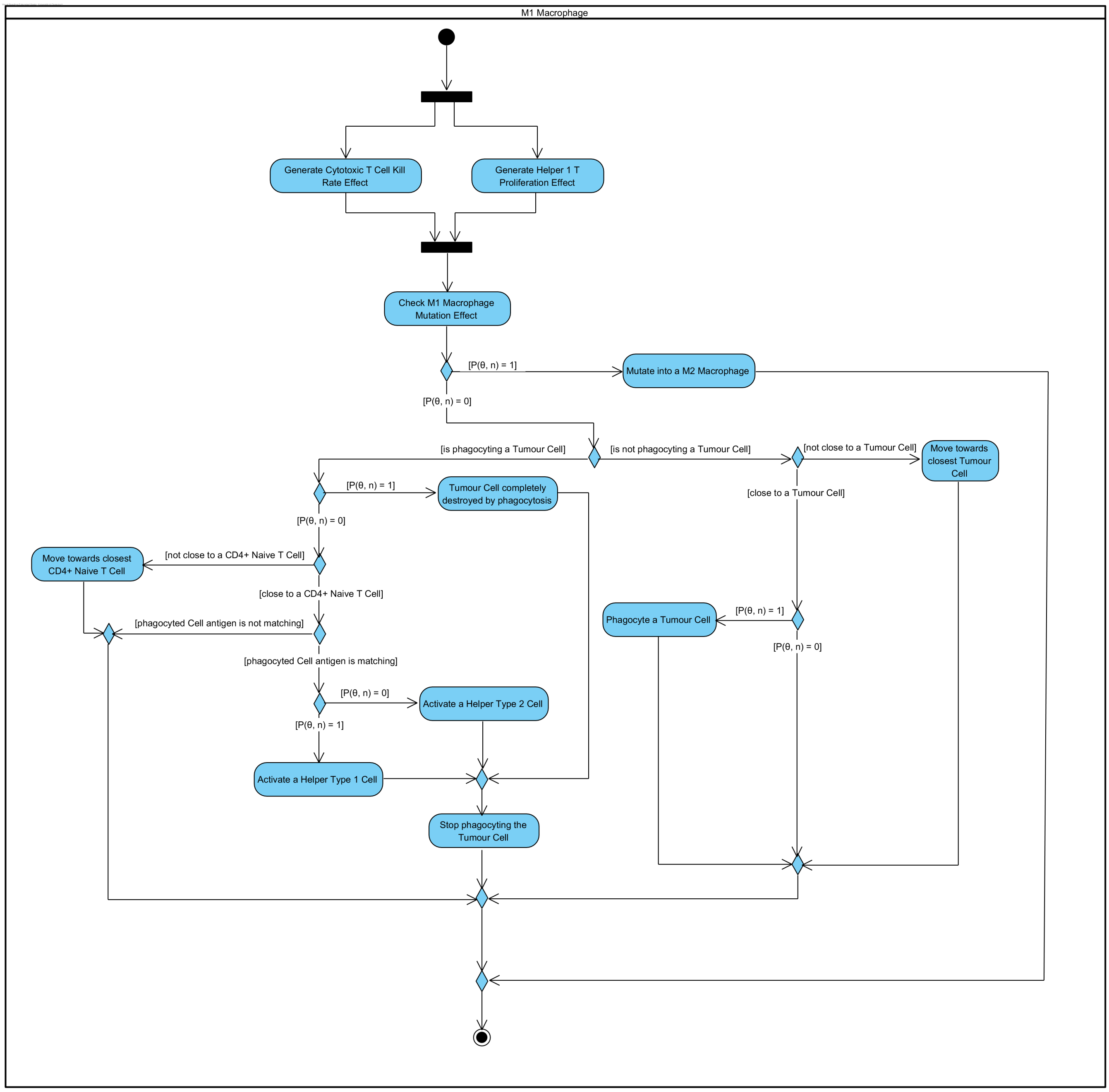

Figure S9: This activity diagram shows the behaviour of an M1 Macrophage at each time step of the simulation since the start of the treatment.

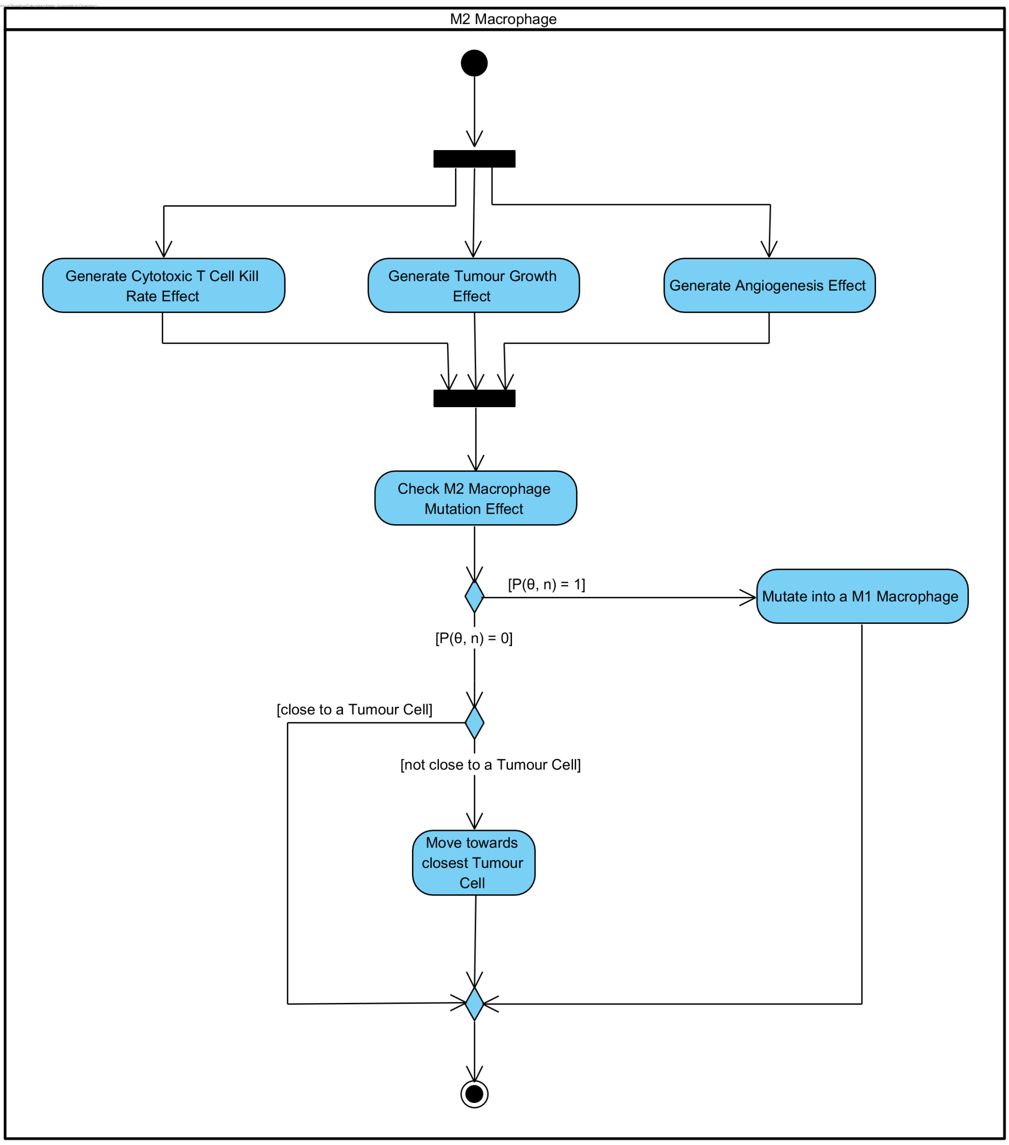

Figure S10: This activity diagram shows the behaviour of an M2 Macrophage at each time step of the simulation since the start of the treatment. The M2 Macrophage exposes a Cytotoxic T Cell Kill Rate Effect, Tumour Growth Effect and Angiogenesis Effect. It has a chance, affected by any present M2 Macrophage Mutation Effect, to mutate into the M1 phenotype. If no mutation occurs, then it will move towards the closest Tumour Cell.

1.4 Mast Cell Agents

Mast cells play a protective role, especially in the defence against pathogens; by producing a compound called histamine, they are able to activate the dendritic cells. Mast cells have also been observed playing a pro-tumorigenic role in some cases, a protective function in others, or even not participating in the cancer processes at all, although present in the tumour proximity (Varricchi et al., 2017). In the case of Renal Cell Carcinoma, it has been reported by Chen et al. (2017) that mast cells indeed foster tumour angiogenesis.

The *mast cell agents* are modelled referring to Varricchi et al. (2017). They perceive and move towards the tumour mass and stick to it. They have a chance to activate new dendritic cell agents,

which appear at the edge of the 3D environment. Moreover, they may have any combination of the following effects: positive `Angiogenesis Effect`, positive `M1 Macrophage Mutation Effect`, positive `Tumour Apoptosis Effect`, negative `Cytotoxic T Cell Kill Rate Effect` and any value of `Tumour Growth Effect`. The subset of these effects that every mast cell agent may have is chosen randomly (with a 50% chance for each effect to be present) when the agent is created.

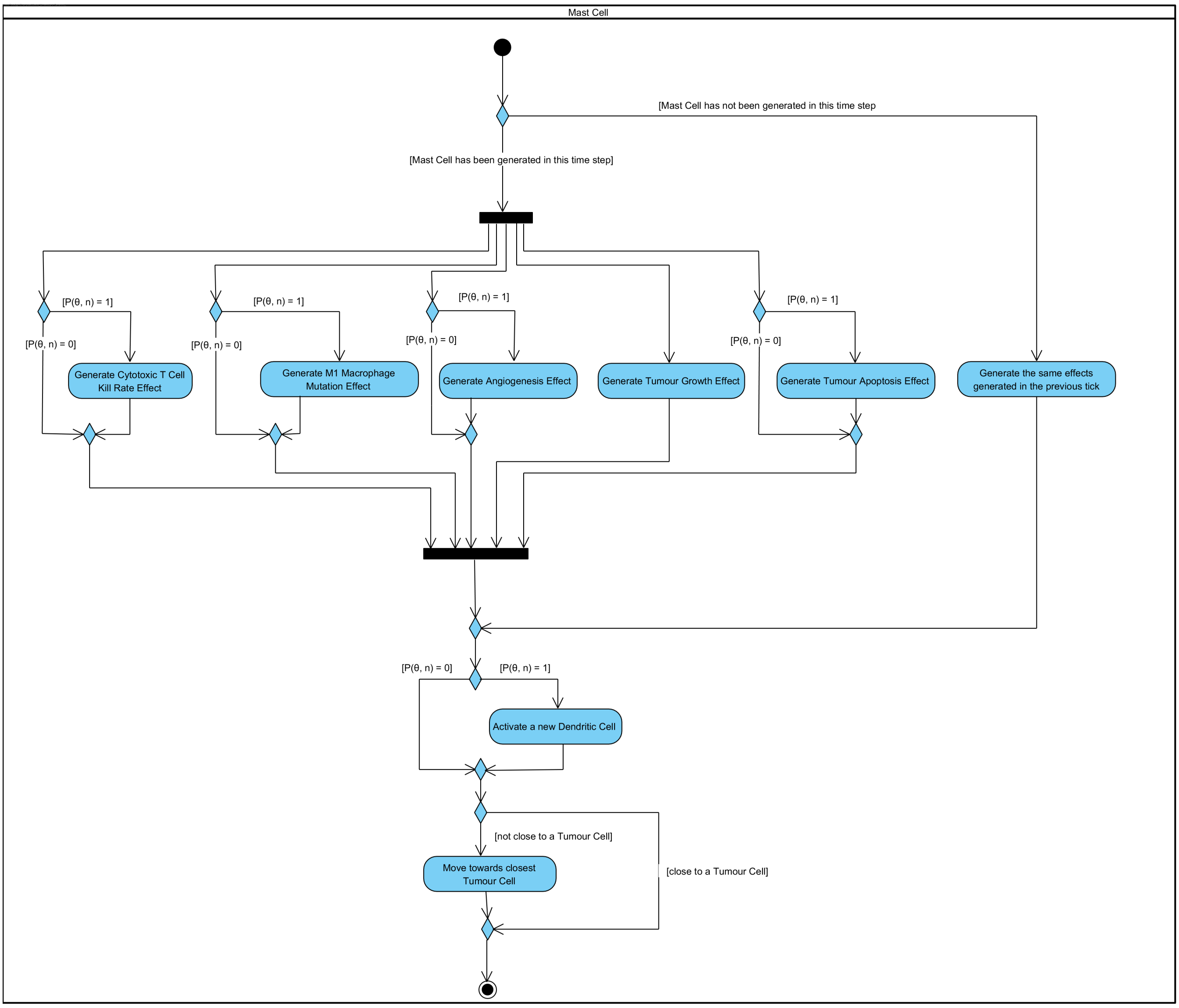

Figure S11: This activity diagram shows the behaviour of a Mast Cell at each time step of the simulation since the start of the treatment. The Mast Cell exposes a Tumour Growth Effect and any combination of the following effects: Cytotoxic T Cell Kill Rate Effect, M1 Macrophage Mutation Effect, Angiogenesis Effect and Tumour Apoptosis Effect. The Mast Cell has a chance to activate a new Dendritic Cell and will move towards the closest Tumour Cell.

1.5 Natural Killer Cell Agents

Natural killer (NK) cells are cytotoxic lymphocytes that are able to kill tumour cells, even in the absence of surface antigens. This role is fundamental in the immune response, since T cells can recognise pathogens only if they present antigenic peptides on their surface (Vivier et al., 2011).

Natural killer cell agents perceive and move towards RCC cell agents and attempt to destroy them; this action has a high probability associated and can be influenced by the `Natural Killer Cell Kill Rate Effect`.

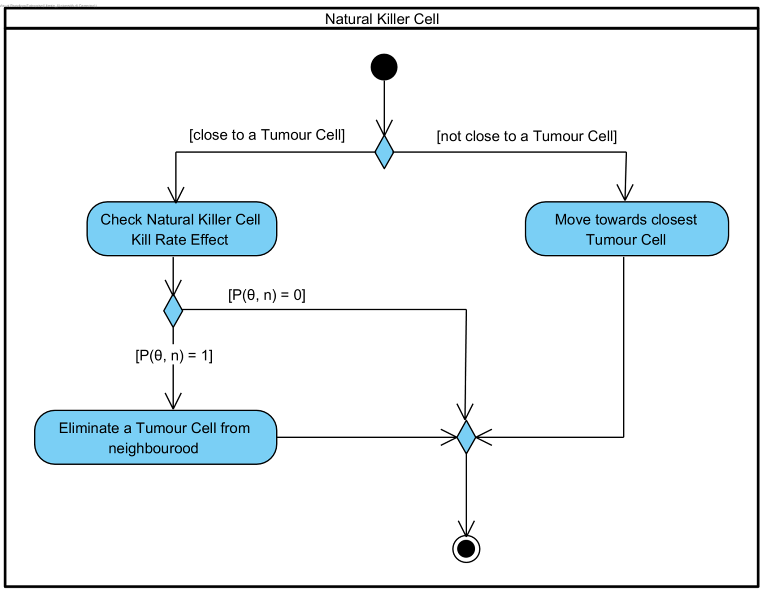

Figure S12: This activity diagram shows the behaviour of a Natural Killer Cell at each time step of the simulation since the start of the treatment. The Natural Killer Cell will move towards the closest Tumour Cell and attempt to destroy it, with the success of this latter action influenced by any present Natural Killer Cell Kill Rate Effect.

1.6 Neutrophil Cell Agents

Neutrophils represent the most abundant type white blood cells and are the first type of inflammatory cells that move towards the site of an inflammation. In the case of Renal Cell Carcinoma, the neutrophil/lymphocyte (LNR) ratio is linearly associated with tumour size (Arda et al., 2018) and an elevated neutrophil count is linked to a poor prognosis (Shen et al., 2019).

However, according to Santoni et al. (2019), the prevalence of neutrophils was observed to be unchanged in both lean and obese patients, and they do not seem to have any relevant behaviour in the context of RCC; therefore, the *neutrophil cell agents* have been modelled as a dummy, meaning that it does not perform any action nor produce any effect.

2 RANDOMNESS AND PROBABILITY

The simulation relies on a random number generator (RNG), which makes agents act in a probabilistic way, and most agents are placed in a random location inside the environment. However, it is possible to save the seed number used to initialise the RNG and repeat the same simulation.

Nonetheless, not all actions are associated with a probability; as an example, agent movement always succeed. When a probability is taken into account, there are usually three elements involved: the *limit value*, the *threshold value* and the *random number* generated. It is useful to imagine the limit value n ($n \in \mathbb{N}, n \geq 1$) as an n-sided die (obviously, with $n = 2$ it is a coin and with $n = 1$ it is a guaranteed result). The random number generated is always between 0 and $n - 1$ included; therefore, it is just like rolling a die with n sides. We also consider a threshold value θ, such that $\theta \in \mathbb{Z}$, which defines when the roll can be considered a success: that is, when the generated value is less than or equal to θ. It should be noticed that we allow threshold values across all integers. This means that, when $\theta < 0$, then $n' = n - \theta$ and $\theta' = 0$; that is, the sides of the imaginary die are increased and the threshold set to 0, thus making a successful roll more difficult ($n' > n$). Every action that needs to be executed probabilistically has its own value of n fixed from the start, while the value of θ, which defaults to 0, may vary every time the action is executed, according to the current effects that are influencing the agent, which are described in Section 3.1 of the article and summarised in Table S2.

The following predicate summarizes the discussion above:

$$P(\theta, n) = \begin{cases} X \sim \mathcal{U}(0,1) < \frac{\theta+1}{n} & \text{if } \theta \geq 0 \\ \\ X \sim \mathcal{U}(0,1) < \frac{1}{n-\theta} & \text{if } \theta < 0 \end{cases}$$

where $\theta \in \mathbb{Z}$, $n \in \mathbb{N}_{>0}$ and X is a random variable drawn from the continuous uniform distribution in the unit interval.

When this predicate is satisfied, that is when $P(\theta, n) = 1$, the action it is associated with succeeds, otherwise, with $P(\theta, n) = 0$, the action fails. The value of θ induces two different cases to consider, one when $\theta \geq 0$ (top row in the equation) and one when $\theta < 0$ (bottom row in the equation). In the former case, the random variable X is compared to the value of $\theta + 1$ over n. Only if the value of X is strictly less than the ratio of $\theta + 1$ over n the predicate is satisfied and is equal to 1. The latter case of the predicate (bottom row of the equation) is evaluated when $\theta < 0$. In this case the random variable X is compared to the ratio of 1 over $n - \theta$, and the predicate is satisfied, as usual, only if X is strictly less than this ratio. Notice that when this case of the predicate is evaluated, with $\theta < 0$, the value $n - \theta$ is always a positive natural number strictly greater than n.

For example, consider an action with associated $n = 5$. Normally, that is with $\theta = 0$, the probability of success for the action is $\frac{1}{5} = 20\%$. If we suppose that some positive effects increase the value of θ to 2, the probability of success will be $\frac{3}{5}$, that is 60%. Some time after that, we may observe a $\theta = -3$, and the probability of success becomes $\frac{1}{8}$, which means 12.5%.

Effect	Description
Angiogenesis Effect	Affects the likelihood for the tumour as a whole to start angiogenesis.
Cytotoxic T Cell Activation Effect	Affects the likelihood to successfully activate a CD8+ naive T cell into a CD8+ cytotoxic T cell.
Cytotoxic T Cell Apoptosis Effect	When positive, enables a chance of a CD8+ cytotoxic T cell to undergo apoptosis (cell death); the greater the value, the greater the likelihood.
Cytotoxic T Cell Kill Rate Effect	Affects the likelihood for a CD8+ cytotoxic T cell to eliminate a tumour cell.
Cytotoxic T Cell Proliferation Effect	Affects the rate of duplication of CD8+ cytotoxic T cells.
Dendritic Cell Phagocytosis Effect	Affects the likelihood for a dendritic cell or plasmacytoid dendritic cell to phagocyte a tumour cell.
M1 Macrophage Mutation Effect	Affects the rate of the M1 macrophages switching into the M2 phenotype.
M2 Macrophage Mutation Effect	Affects the rate of the M2 macrophages switching into the M1 phenotype.
Natural Killer Cell Kill Rate Effect	Affects the likelihood for a natural killer cell to eliminate a tumour cell.
Regulatory T Differentiation Effect	Affects the rate of CD4+ naive T cells differentiation into regulatory T cells.
Helper 1 T Proliferation Effect	Affects the rate of duplication of CD4+ helper 1 T cells.
Tumour Apoptosis Effect	When positive, enables a chance of a tumour cell to undergo apoptosis (cell death); the greater the value, the greater the likelihood.
Tumour Growth Effect	Affects the rate of duplication of tumour cells.

Table S2. The effects modelled in the system.

REFERENCES

Arda, E., Yuksel, I., Cakiroglu, B., Akdeniz, E., and Cilesiz, N. (2018). Valuation of neutrophil/lymphocyte ratio in renal cell carcinoma grading and progression. *Cureus* 10

Carretero, R., Sektioglu, I. M., Garbi, N., Salgado, O. C., Beckhove, P., and Hämmerling, G. J. (2015). Eosinophils orchestrate cancer rejection by normalizing tumor vessels and enhancing infiltration of cd8+ t cells. *Nature Immunology* 16, 609–617

Celada, F. and Seiden, P. (1992). A computer model of cellular interactions in the immune system. *Immunology Today* 13, 56–62

Chen, Y., Li, C., Xie, H., Fan, Y., Yang, Z., Ma, J., et al. (2017). Infiltrating mast cells promote renal cell carcinoma angiogenesis by modulating pi3k-akt-gsk3beta-am signaling. *Oncogene* 36, 2879–2888

Dandekar, R. C., Kingaonkar, A. V., and Dhabekar, G. S. (2011). Role of macrophages in malignancy. *Annals of Maxillofacial Surgery* 1, 150–154

Kim, H.-J. and Cantor, H. (2014). Cd4 t-cell subsets and tumor immunity: The helpful and the not-so-helpful. *Cancer Immunology Research* 2, 91–98

Koucký, V., Bouček, J., and Fialová, A. (2019). Immunology of plasmacytoid dendritic cells in solid tumors: A brief review. *Cancers* 11, 470

Lin, W.-W. and Karin, M. (2007). A cytokine-mediated link between innate immunity, inflammation, and cancer. *The Journal of Clinical Investigation* 117, 1175–1183

Mailliard, R. B., Egawa, S., Cai, Q., Kalinska, A., Bykovskaya, S. N., Lotze, M. T., et al. (2002). Complementary dendritic cell–activating function of cd8+ and cd4+ t cells. *Journal of Experimental Medicine* 195, 473–483

Ohue, Y. and Nishikawa, H. (2019). Regulatory t (treg) cells in cancer: Can treg cells be a new therapeutic target? *Cancer Science* 110, 2080–2089

Santoni, M., Cortellini, A., and Buti, S. (2019). Unlocking the secret of the obesity paradox in renal tumours. *The Lancet Oncology*

Schwaab, T., Schned, A., Heaney, J., Cole, B., Atzpodien, J., Wittke, F., et al. (1999). In vivo description of dendritic cells in human renal cell carcinoma. *Journal of Urology* 162, 567–573

Shen, J., Chen, Z., Fan, M., Lu, H., Zhuang, Q., and He, X. (2019). Prognostic value of pretreatment neutrophil count in metastatic renal cell carcinoma: a systematic review and meta-analysis. *Cancer Management and Research* 11, 5365—5374

Varricchi, G., Galdiero, M. R., Loffredo, S., Marone, G., Iannone, R., Marone, G., et al. (2017). Are mast cells masters in cancer? *Frontiers in Immunology* 8, 424

Vivier, E., Raulet, D. H., Moretta, A., Caligiuri, M. A., Zitvogel, L., Lanier, L. L., et al. (2011). Innate or adaptive immunity? the example of natural killer cells. *Science* 331, 44–49

Vlahopoulos, S. A. (2017). Aberrant control of nf-kb in cancer permits transcriptional and phenotypic plasticity, to curtail dependence on host tissue: molecular mode. *Cancer Biology & Medicine* 14, 254–270
